# Yes, no, maybe so: the importance of cognitive interviewing to enhance structured surveys on respectful maternity care in northern India

**DOI:** 10.1093/heapol/czz141

**Published:** 2019-10-31

**Authors:** Kerry Scott, Dipanwita Gharai, Manjula Sharma, Namrata Choudhury, Bibha Mishra, Sara Chamberlain, Amnesty LeFevre

**Affiliations:** 1 Department of International Health, Johns Hopkins Bloomberg School of Public Health, 615 N. Wolfe Street, Baltimore, MD 21218, USA; 2 Oxford Policy Management, New Delhi, India; 3 Independent Consultant, New Delhi, India; 4 BBC Media Action, New Delhi, India; 5 Health Intelligence Initiative, Division of Epidemiology and Biostatistics, School of Public Health and Family Medicine, University of Cape Town, Cape Town, South Africa

**Keywords:** India, respectful maternity care, disrespect and abuse, patient–provider relationship, cognitive interviews, survey design

## Abstract

Quantitative survey findings are important in measuring health-related phenomena, including on sensitive topics such as respectful maternity care (RMC). But how well do survey results truly capture respondent experiences and opinions? Quantitative tool development and piloting often involve translating questions from other settings and assessing the mechanics of implementation, which fails to deeply explore how respondents understand survey questions and response options. To address this gap, we conducted cognitive interviews on survey questions (*n* = 88) adapted from validated RMC instruments used in Ethiopia, Kenya and elsewhere in India. Cognitive interviews with rural women (*n* = 21) in Madhya Pradesh, India involved asking the respondent the survey question, recording her response, then interviewing her about what the question and response options meant to her. We analysed the interviews to revise the tool and identify question failures, which we grouped into six areas: issues with sequencing, length and sensitivity; problematic response options; inappropriate vocabulary; temporal and spatial confusion; accessing different cognitive domains; and failure to resonate with the respondent’s worldview and reality. Although women tended to provide initial answers to the survey questions, cognitive interviews revealed widespread mismatch between respondent interpretation and question intent. Likert scale response options were generally incomprehensible and questions involving hypothetical scenarios could be interpreted in unexpected ways. Many key terms and concepts from the international RMC literature did not translate well and showed low resonance with respondents, including consent and being involved in decisions about one’s care. This study highlights the threat to data quality and the validity of findings when translating quantitative surveys between languages and cultures and showcases the value of cognitive interviews in identifying question failures. While survey tool revision can address many of these issues, further critical discussion is needed on the use of standardized questions to assess the same domains across contexts.

Key Messages

- Cognitive interviewing assesses whether quantitative survey questions access the intended cognitive domain among respondents; we applied this research methodology to a survey on respectful maternity care (RMC) in rural India that had been validated in other contexts and translated into Hindi.
- Women’s initial responses to the survey questions frequently elicited answers with no bearing on the question’s intent, at times due to cognitive mismatch wherein respondents interpreted questions drastically differently than the researchers intended.
- Likert response options were incomprehensible to most respondents and a number of RMC concepts, such as consent and being involved in decisions about one’s care, failed to resonate with women’s worldviews and realities.
- Without careful testing and adaptation to local contexts, survey findings used to inform public health policy are at risk of inaccurately representing respondents’ experiences and opinions.

## Introduction

Respectful maternity care (RMC) is defined by the World Health Organization as ‘care organized for and provided to all women in a manner that maintains their dignity, privacy and confidentiality, ensures freedom from harm and mistreatment, and enables informed choice and continuous support during labour and childbirth’ (WHO, 2018). Despite widespread recognition of the importance of RMC as a health systems and human rights imperative (D’Oliveira *et al*., 2002; Sen *et al*., 2018), there is limited quantitative evidence on rates of disrespect and abuse and there is wide variation in how domains of RMC are conceptualized. Bohren *et al*.’s (2015) review identified 12 studies with relevant quantitative data but only 3 that explored mistreatment of women during childbirth in health facilities as a primary objective. Reports of any mistreatment ranged from 15% (Sando *et al*., 2014) in Tanzania to 98% in Nigeria (Okafor *et al*., 2015). The most prevalent types of abuse (reported by >20% of respondents in at least one of the three studies) were: non-confidential care, lack of physical privacy, non-consented interventions, detention in facilities, denial of birth companionship, neglect and abandonment, discrimination based on patient attributes, unclean facilities and physical abuse. Furthermore, while the body of research on women’s experiences across the continuum of maternity care, including during antenatal care, is growing, there remain many gaps (Bohren *et al*., 2015).

The routine and comprehensive measurement of women’s experiences during maternity care, including RMC, is vital to improving quality of care and in turn maternal and child health outcomes (Knight *et al*., 2013; Nair *et al*., 2014). Measurement allows the severity and nature of the issue to be assessed and changes over time to be tracked. The development of structured survey tools requires assessment of their validity and reliability for local populations in regional languages. The process often starts with a review of the literature, then involves the generation of indicators, drawing from existing data collection tools and expert inputs, and culminates with pilot testing. Pilot testing most routinely focuses on the mechanics of survey implementation by enumerators and may include efforts to assess the tool’s length, skip patterns, and response options and remedy obvious errors in translation. Methods may also include the observation of how enumerators engage with respondents with the broader aim of improving how the tool is implemented. However, there is often limited exploration of the extent to which survey questions truly measure what the researchers intend them to, particularly among populations with different experiences and worldviews from the researchers.

Cognitive interviewing is an often-overlooked research methodology used to qualitatively assess and improve the cognitive match between a quantitative survey question’s intent and the respondent’s interpretation and response. It can be defined as ‘the administration of draft survey questions while collecting additional verbal information about the survey responses, which is used to evaluate the quality of the response or to help determine whether the question is generating the information that its author intends’ (Beatty and Willis, 2007). Applications to date include understanding the thought processes and cognitive biases that underpin quality-adjusted life-year determinations (Patenaude and Bärnighausen, 2019) as well as assessing and adapting quantitative survey tools on topics ranging from the use of alternative and complementary medicine (Esteban *et al*., 2016), satisfaction with home care (Aletras *et al*., 2010) and physical activity (Finger *et al*., 2015). Cognitive interviews often include assessing the quality of translations (Farage *et al*., 2012; Hall *et al*., 2013; Zeldenryk *et al*., 2013) but extend beyond word choice to understanding whether respondents interpret question concepts as intended by researchers. In the context of measuring RMC, cognitive interviewing has the potential to offer important insights into a survey’s success at linguistic and cultural translation of global domains (Bowser and Hill, 2010), as well as comprehensibility and suitability for the respondent group.

This manuscript presents findings from cognitive testing of an RMC measurement tool for use in rural northern India. We explain the methodological process, present a typology of problems identified, and explain how we altered our RMC survey tool based on this process. We end with broader reflections on the difficulties associated with using quantitative surveys in populations unaccustomed to this type of interaction and on measuring internationally determined domains of RMC among diverse populations.

## Methods

### Study setting

The study took place in Madhya Pradesh, a Hindi-speaking state in central India, which has a population of 75 million. While almost 90% of households have some (often limited and irregular) access to electricity, only 34% have improved sanitation (MoHFW, 2017). Male literacy is 82% and female literacy is 59%. In 2015, 53% of pregnant women received antenatal care (ANC) in the first trimester, 36% received the recommended four ANC visits and 81% delivered in a health facility (MoHFW, 2017).

### Tool development

Structured questionnaires were developed to assess women’s RMC during pregnancy and childbirth across internationally recognized domains (failure to meet professional standards of care, health system conditions and constraints, poor rapport between women and providers, verbal abuse, physical or sexual abuse, stigma and discrimination; Bohren *et al*., 2015). Items were generated from validated surveys developed in Ethiopia (Sheferaw *et al*., 2016) and Kenya (Afulani *et al*., 2017), as well as survey tools used in northern India by other research teams. Our interest in conducting cognitive interviews arose from our desire to assess the face validity of the survey instrument, because it drew from a range of sources.

The pregnant women’s tool was comprised of 37 questions pertaining to women’s experiences during ANC. The post-partum women’s tool was comprised of 51 questions about treatment during labour and delivery (first columns of Supplementary Table S1). Questions in the pregnant women’s tool were a sub-set of the questions for post-partum women, adjusted slightly for relevance to ANC (e.g. where the post-partum question asked ‘At any point during your stay for this delivery were you physically harmed by any of the health care workers?’ the ANC question asked ‘At any point during your most recent ANC visit, were you physically harmed by any of the health care workers?’).

**Figure 1:**
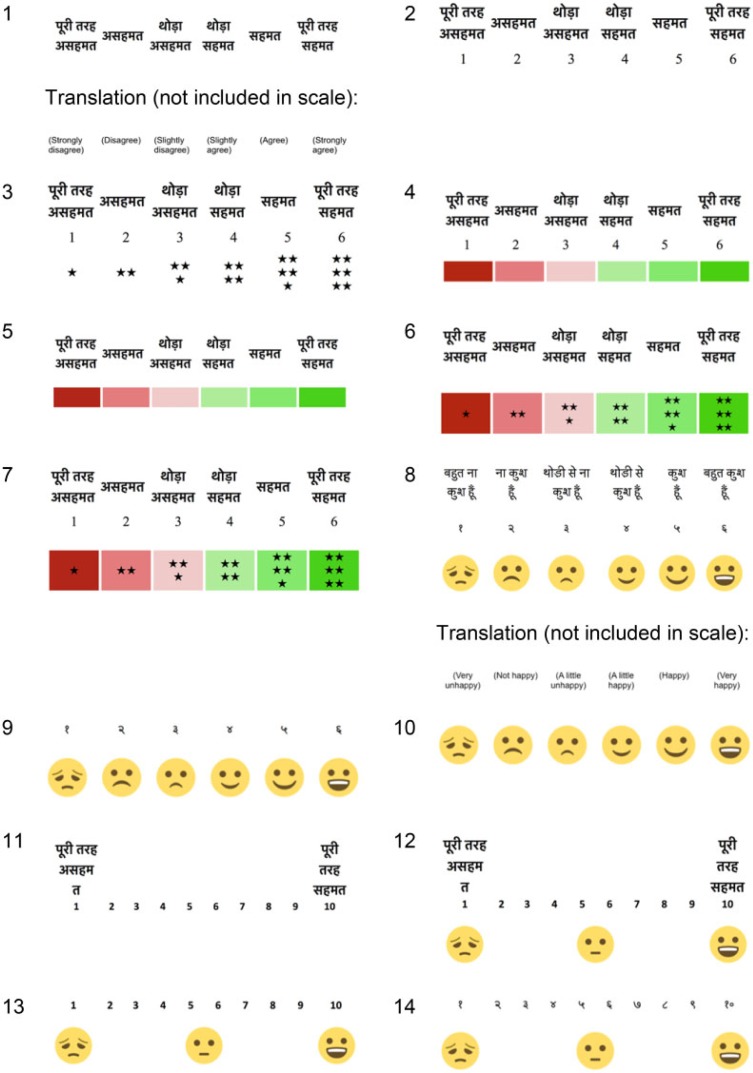
Likert scales developed for cognitive testing.

The questions were translated into Hindi by a professional Delhi-based translator. Among the total of 88 questions (37 in the pregnant women’s tool and 51 in the post-partum tool), 42 were Likert questions wherein respondents were read a statement (e.g. ‘I would recommend the place of my most recent ANC visit to other women’) and asked to indicate their level of agreement. To explore optimal Likert response options, we developed 14 Likert scale response options (Figure 1). These included six-point scales (strongly agree, agree, somewhat agree, somewhat disagree, disagree or strongly disagree) and 10 point scales (from 10 ‘strongly agree’ to 1 ‘strongly disagree’), English and Hindi numerals, and various combinations of numbers, colours, stars, words and smiley face emoji. We also tried using happiness (very happy to very unhappy) rather than agreement for some questions.

### Cognitive interviews

Cognitive interviews were conducted in February, March and September 2018 with pregnant and post-partum women. We undertook three rounds of tool revision (Willis, 2004): the original version of the tool was revised after the first round of interviews; this second version was tested and revised again; the third version was tested and underwent a final round of revisions to produce the final version. Interviews were carried out by four experienced, native Hindispeaking female qualitative researchers (co-authors D.G., M.S., N.C. and B.M.) with master’s level degrees in the social sciences, who received 2 days of training. They conducted the cognitive interviews using the verbal probing approach (Willis and Artino, 2013), by following an interview guide that listed the quantitative survey questions and answer options, and, after each one, a series of qualitative questions and probes to assess cognitive match between the question’s intent and the respondent’s interpretation and answer (Box 1).

Box 1 Example section of the cognitive interview guide.

Question 1. ‘When you went for delivery to the health facility did the doctors, nurses, or other health care providers introduce themselves to you when they first came to see you?’

Response options: yes, no, don’t know, no response

Cognitive probes:

- What happened when you first met the doctors, nurses or other healthcare providers?
- What did they say to you?
- Can you tell me about how they spoke to you when you arrived? What kinds of things did they say? How did they sound—speaking with love/nicely or harshly?
- What does ‘introduction’ [parichay] mean to you? What other word could we use?
- Did you find this question easy to understand/answer? Was it easy to remember what happened?

For each question, interviewers were instructed to read the exact survey question, attempt to elicit an answer according to the answer options available, and then interview the respondent about the question and her related experience, using the probes provided as required. The three most common probes were ‘Can you tell me what happened?’; ‘What does [key term from the survey question] mean to you?’ and ‘Why did you say [survey answer]?’ Qualitative questions and probes invited the respondent to share a narrative description of the event, explored their understanding of key vocabulary words (including identifying alternative words used in the area), and asked them to explain why they gave their chosen response option. The interviewers were invited to use the pre-developed ‘proactive verbal probes’ as appropriate and to create and use their own ‘reactive verbal probes’ during the interview according to their assessment of the respondent’s interpretation (Willis and Artino, 2013). Another common cognitive interviewing methodology—the ‘think aloud’ method (Willis and Artino, 2013)—was attempted but quickly abandoned. When we asked our first three respondents to verbalize their thoughts while considering a survey question and determining which answer to provide they struggled to understand our request and could not think of anything to say.

For the Likert style questions, the interviewers explained that they were going to read a statement and ask the respondent for her level of agreement, then showed one of the Likert scales. Before beginning, they explained the scale by reading each level of agreement (on the 6-point scale) or the extreme ends of the scale (on the 10-point scale) while pointing to each place on the scale and explaining that the respondent could indicate which point was most appropriate. The interviewer then read the question and again repeated the response options while pointing to them on the Likert scale. She then asked the respondent to indicate the most appropriate place on the scale. The interviewer finally interviewed the respondent about how she understood the statement and why she selected the response option provided (in cases where a response was forthcoming). For additional Likert style questions, the interviewers tried new Likert response scales. In some cases, multiple scales were shown and explained for the same question. All the scales were tested with at least four respondents.

### Sample

We sampled 21 rural women living one to 3 hours from the cities of Indore and Bhopal in Madhya Pradesh (Table 1). For the cognitive interviews on the pregnant women’s RMC survey tool, we recruited pregnant women who received ANC within the preceding 2 months. For the post-partum RMC survey tool, we recruited women who delivered in a healthcare facility within the last 4 months. As the survey questions on RMC during ANC were essentially a subset of the questions for post-partum women, we decided to sample a larger number of post-partum women.

**Table 1:** Respondent sample

	Original tool	Revised tool 1	Revised tool 2	Total
Post-partum women	8	4	3	15
Pregnant women	4	1	1	6
Total	12	5	4	21

After receiving permission from the national- and state-level governments, respondents were identified through first approaching the local auxiliary nurse midwife and then the village-based ASHA community health worker or Anganwadi worker, who introduced us to women in their catchment areas who fit these profiles. The researchers then approached women and invited them to participate in an interview about their recent ANC or delivery. If the woman agreed, the interviews were arranged to take place in respondents’ homes at a convenient time. We worked with the health workers to ensure respondent diversity by education, affluence and caste. Six were highly marginalized (five or fewer years education, tribal or low caste, with few assets and unfinished mud floor homes), four women had 12 or more years’ education, and two were higher caste and affluent. Almost half the respondents were pregnant with or had recently given birth to their first child, while three were pregnant with or had recently given birth to their third or fourth child. All the women were homemakers, farmers or daily wage labourers and were aged between 21 and 29.

### Data collection

Interviews commenced after reading the consent form, reiterating its contents in colloquial language and receiving verbal informed consent. Respondents were given the option of recording—all but one agreed. During the interview, the interviewers and accompanying note-takers wrote observations about the respondent’s response, circled or underlined words and phrases in the survey questionnaire that caused issues, and jotted down alternative vocabulary, helpful examples or other suggestions that arose.

### Analysis

The team regrouped daily (after four to eight interviews) for detailed discussion of issues that had arisen. Potential revisions were drafted and at two points in the process we revised the quantitative survey questions (re-phrased survey questions, amended response options, revised question order) for further testing, with new cognitive interview probes as needed. The audio recordings of the interviews were transcribed and translated into English. The researchers read the transcripts while listening to the audio recordings to ensure translation quality, including the retention of all key Hindi words. The transcripts and notes were then read to generate a typology of question failures and the interviews were coded against the typology. Finally, a subset of the five richest audio recordings of the cognitive interviews using the original tool was listened to again and question failures were checked against the typology.

## Findings

We first present broad summary findings and then specific findings structured according to our typology of survey question failures.

Cognitive interviews revealed extensive and largely unanticipated question failures in the initial RMC tool. These question failures resulted in an overall survey that was often incomprehensible to the respondents and that frequently failed to measure the intended RMC domains. These issues have been grouped into a typology of six question failures, developed inductively through debriefs, team discussions, and detailed examination of the data (Table 2). After two rounds of revision based on these cognitive interviews, the final tool achieved appropriate length and sequencing, and showed improvement in terms of response options, comprehensibility, cognitive match and resonance with local worldviews and realities. Please see Supplementary Table S1 for the original and revised questions in English and Hindi, as well as notes on the changes.

**Table 2:** Typology of RMC survey question failures, identified through cognitive interviews

Question failure type	Explanation	Example (see text and Supplementary Table S1 for more examples)
1. Issues with sequencing, length and sensitivity	Question order does not flow well, sensitive questions come too early (before there has been time to establish adequate rapport), respondents find the tool long and/or repetitive.	Questions about physical abuse during pregnancy were initially placed early in the survey before sufficient rapport was established, which made respondents uncomfortable and unlikely to disclose negative experiences.
2. Problematic response options	Response options fail to capture frequent replies are inappropriate, or are confusing to respondents.	Likert scales and the concept of graduations of agreement or disagreement along a spectrum were incomprehensible to most respondents and failed to capture meaningful responses.
3. Inappropriate vocabulary and long sentences	Key vocabulary terms not locally understood; long sentences and sentences with multiple components are difficult for respondents to follow.	The initial translations of keywords such as delivery, health centre, physically harmed, sterilization, insurance, vaginal and many others were not understood.
4. Temporal and spatial confusion	Mismatch between the time and location that the survey question was seeking to assess and the time and location that respondents considered.	When a respondent was asked whether she was allowed to drink liquids or eat any food while in labour she replied ‘yes’. Upon probing she told us about the nutrition advice she received from the health facility staff throughout her pregnancy.
5. Accessing different cognitive domains	Question accesses a different cognitive domain than was the interviewer and question developer’s intent.	A respondent replied that she would return to the same place of delivery in the future; but on probing we found that she was thinking about her inability to afford healthcare elsewhere (in a private facility) rather than her satisfaction with the services she received.
6. Failure to resonate with the respondent’s worldview and reality	Question asks about a domain of global importance that does not align with local assessments of respectful care.	Respondents expected healthcare providers to use their knowledge and experience to make decisions about the best course of action for the woman and her baby; women did not understand the idea of being involved in decisions about their care.

Before presenting findings on the specific question failures, several initial observations are salient. First, respondents tended to provide a response to the questions. However, upon probing, we frequently found that the answer given was not linked to the question at hand and was often the exact opposite to what the respondent experienced. This issue was caused by respondents having either not understood the question at all and simply wanting to move on or having understood the questions in a different manner than was the survey’s intent. For instance, a respondent (CT_PP_09), when asked whether the staff asked permission/consent before doing procedures and examinations first said, ‘yes’. The interviewer followed up with ‘how did they ask?’ to which the respondent replied, ‘They were doing the procedures but they did not ask me’. Another (CT_PP_08) replied ‘yes’ to the question about whether her body was covered by cloth, curtains or a screen so that outsiders could not see her in labour. Upon additional discussion it was clear that she was uncovered and no screens were used, such that all six other women labouring in the room as well as their attendants could see her. In another interview (CT_PP_15), the respondent immediately replied ‘yes’ to the question on whether the health facility staff introduced themselves to her when she arrived at the facility. When asked about the interaction, the respondent first replied that she did not understand the word ‘introduction’ [parichay] and, after the interviewer explained the concept, the respondent clarified that no greeting or introduction had taken place. In a typical survey, these initial responses would have been recorded as final and the researchers would never know that the question was failing to measure its intended construct.

Second, despite repeated attempts by researchers to explain that the interviews were aiming to improve survey questions for future use with other women, respondents appeared to understand the interaction solely as an interview about their recent maternity care experience, and a related test of their memory, knowledge or vocabulary.

I Will other people understand if we will use this word [ukhru bethna, i.e., squatting position]?

[No response.]

I Are you understanding? I am talking to you first, then I will speak to other people later. If I will tell them that you were sitting ukhru [squatting] –

R No, but delivery didn’t happen in this way. (CT_PP_01)

I So, like I am asking you about physical hurt and you had trouble understanding. What should I have asked for you to understand more easily? How should I have asked? … If you tell me, when I talk to the other women it will be easier for me to talk to them, I’d be able to explain things better.

R No madam, nothing like that happened to me… No one scolded me (CT_PP_04).

Very few respondents engaged with the cognitive interview process from a macro perspective. Macro level engagement could have been indicated by respondents suggesting improved wording, explaining how other women in their community might interpret a question, or reflecting verbally on the larger project of developing a successful quantitative survey tool. Despite extensive efforts by the researchers to explain the intention of the interviews, respondents sometimes appeared anxious, as if they were being tested and were failing when they could not understand questions or response options.

### Question failure type 1: Sequencing, length and sensitivity

Respondents and interviewers expressed discomfort with the initial sequence of questions, which asked about verbal and physical abuse within the first few questions. These questions had been placed early in the survey because they were key indicators that researchers wanted to gather data on before any risk of respondent fatigue. However, these questions were too sensitive to come early in the survey and were shifted to the end, after rapport was established.

Both respondents and interviewers found the initial survey to be excessively long as well as repetitive. The original survey sought to measure both prevalence and satisfaction, with prevalence assessed through questions about whether an event occurred (e.g. At any point during your stay for this delivery were you left un attended by health providers when you needed care? Yes/no response options) and satisfaction assessed through Likert questions (e.g. I was left alone in the facility when I needed assistance from the doctors, nurses or other healthcare providers. Six response options, from strongly agree to strongly disagree). However, respondents did not register a difference between these questions.

There were some questions that respondents—and researchers— found inappropriate. For example, when asking about physical abuse, the original question had probes for specific types of abuse including rape. In the original question that asked the respondent to speculate on the possible reasons for physical or verbal abuse, one possible reason was ‘your sex’, translated to ‘that you are a woman’ [*aapka mahila hona*]; this possible reason for discrimination was non-sensical to respondents because being female is a prerequisite for requiring maternal healthcare and the option was removed. Other problematic components included asking if a woman’s father or father-in-law was in the room with her while she delivered, as it was considered inappropriate for these men to attend a birth.

### Question failure type 2: Problematic response options

None of the respondents who were interviewed using the original tool found the Likert response options comprehensible, despite extensive efforts by the researchers to explain the questions and tying multiple scales per respondent (Box 2). Most respondents did not understand the concept of gradated levels of agreement to a statement or placing agreement or happiness on a scale. The use of colour (shades of red to shades of green), numbers (Hindi or English), 6- or 10-point, smiley faces or different words (agree to disagree, happy to unhappy) did not bring clarity. The failure of the Likert style questions and response options scales was so apparent and uniform across the 12 respondents interviewed using the original survey questions that we removed the Likert questions from the revised tool.

Box 2 Examples of Likert response option failures.

Example 1. Respondent selects smiley face scale response based on her emotional state at the time, rather than the issue in question

For the question: ‘The doctors, nurses or other health care providers at the facility did everything they could to help control my pain’ a respondent pointed to the saddest face [left-most] in the Likert scale below:

**Figure:**
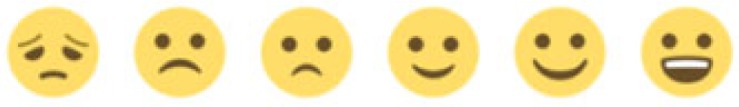

On probing she explained that the staff were supportive and spoke nicely to her, but that since she was in pain her face was ‘like that’ so she selected the saddest face.

Example 2. Respondent does not engage with Likert scale and instead provides a dichotomous response

Many respondents avoided engaging with the Likert scales, instead continuing to repeat their response in terms of a dichotomous yes/no, agree/disagree, happened/did not happen, etc. For example, in response to the statement about procedures being explained before they were conducted, a respondent (CT_PP_05) repeated ‘they didn’t explain’ several times while the interviewer attempted unsuccessfully to get her to convert this response into a Likert option.

Example 3. Respondent selects a response based on how she felt about the information or situation referred to by the statement, rather than her level of agreement with the statement

In some cases, the respondent selected a Likert scale response but was indicating how she felt about the information or situation, rather than her level of agreement with the statement. For example, when responding to the statement ‘The results of examinations were explained to me’ a respondent (CT_PP_05) replied that she agreed ‘a little bit’, which the researchers initially thought could be transferred to the Likert scale as ‘somewhat agree’. However, she was not reporting that only some results were explained to her. Instead, she selected ‘a little bit’ because the result of the examination was negative, as conveyed in the conversation below.

R: They told me. I mean, they told me. There was an issue. Cord was stuck on the baby’s neck, and they told me.

I They told you that. But do you completely agree, or a little bit?

R A little bit.

I Why?

R It was in the baby’s neck. I wasn’t happy. (CT_PP_05)

Respondents would often convert the Likert statement into a question and then reply with ‘yes’ or ‘no’, without engaging with the scaled response options. In a quantitative survey situation, many of these responses would have been transferred into the data collection instrument as strongly disagree or strongly agree, essentially using only the extreme ends of the scale, despite the fact that the respondent was not indicating degrees of agreement or disagreement.

Pro-actively offering a sometimes/somewhat option (rather than just yes or no) enabled more sensitive and appropriate responses for some questions. For example, when asked, ‘Were the health facility washrooms very clean, somewhat clean/somewhat dirty, or very dirty?’ respondents appeared more willing to indicate ‘somewhat clean/somewhat dirty’ than when asked the yes/no question ‘Were the health facility washrooms clean?’, which tended to result in a ‘yes’. With the sometimes/somewhat option, respondents are able to indicate the presence of a problem without taking an extreme stance.

Initially, respondents were asked yes/no ‘screener’ questions about whether they had been physically or verbally abused, then, if they indicated ‘yes’, were asked a series of probes about the type of abuse. However, we found that asking all respondents about whether they experienced various types of abuse generated more complete responses because it ensured closer alignment between respondent and researcher understandings of what was considered abuse. For instance, a respondent (CI_PP_02) was asked whether she was physically harmed and replied ‘no’. However, upon probing, she explained that one nurse gave injections extremely roughly. The initial survey would have counted her as someone who did not experience physical abuse and skipped the question on specific types of physical abuse, thus failing to capture the rough treatment she experienced.

### Question failure type 3: Inappropriate vocabulary and long sentences

The original translations used many words that were unfamiliar to respondents. This issue was typified by one respondent’s request to ‘ask me in Hindi’ (CT_PP_04). Although the questions were entirely in Hindi, the words used were so unfamiliar to her that she thought the interviewer must be using a different language. There were simple issues where the selected word was too academic or simply unfamiliar and could be replaced with an alternative. In many cases, however, a word that was unknown to one respondent was understood by another, and the alternative word that improved comprehension for one woman was unknown to another. In these cases, we decided to list two or more words that enumerators could try, in order to find the one that worked for each individual. There were also a number of keywords with no ideal locally understood alternatives, necessitating the use of examples in the question to attempt to clarify meaning (Table 3).

**Table 3:** Vocabulary issues and potential resolutions

Issue	Original	Improved alternative
Anglicized words more familiar than academic/sanskritized Hindi words	Swasthya kendra [health centre] Prasav [delivery] Sahamati [consent]/anumahati [permission]	Aaspital [hospital] Dilivri [delivery] Parmishin [permission]
Academic/sanskritized words unknown, simple or common Hindi words more widely used	Sharirik roop se [bodily/physically] Udaaharan ke liye [for example] Parichay [introduction]	Can avoid the word by asking if anyone hurt you—respondents assume we mean physically hurt Jaise [like]/matlab [meaning] Aapse jaan-pahachaan [your acquaintance]/naam bataayaa [told you their name]/haalchaal poochi [asked your wellbeing]
Different words understood by different women, thus multiple options provided	Guptang [genital] Ukadoon baithana [squat]	Yoni [vagina]/ batcha hone wali jagah [baby place] Ghutane mod ke [knee bent]/ ukadoon baithana [squat]/ toilet letareen karate samay jaise baithe hai [sitting on toilet]
Keyword not understood. The use of examples aided comprehension.	During your hospital stay, did health providers ever discuss your personal **private [neejith]** health information in a way that others could hear? Did the doctors, nurses or other staff at the facility ask your **consent [sahamati]** before doing your examinations?	Did health providers ever discuss your personal **private [neejith]** health information in a way that others could hear? For example, healthcare providers could say the results of your lab reports loudly, so that others might hear it. Before doing the vaginal exam or any other exam, did the health workers ask you if they could do it / ask permission [parmishan]?

In addition to vocabulary issues, we noticed that the questions were often quite long in Hindi making it difficult for respondents to keep track of the purpose of the sentence. The issue often arose because questions included the clauses, ‘when you were at the health facility for your delivery…’ or ‘at your most recent ANC visit…’ before the core section. Unfortunately, as discussed next, we found that removing these contextualizing components led to confusion about which time, place and actors the question referred to. We attempted to break questions into shorter sentences wherever possible.

### Question failure type 4: Temporal and spatial confusion

There was often a mismatch between time and location that we were seeking to assess and the time and location that respondents considered. While we were seeking only to learn about a woman’s experience during her more recent ANC visit or her time at the health facility for delivery, respondents often considered other interactions with health workers or other periods of time. For ANC, respondents sometimes, upon probing, were found to be considering an earlier interaction with health workers, such as when taking another child for immunization. When asking about delivery, respondents sometimes spoke of their entire pregnancy or of earlier ANC interactions.

Despite wanting to shorten questions by removing clauses such as ‘During your time at the hospital for delivery…’ we decided to retain these components at frequent intervals during the survey. Nonetheless, this temporal confusion occurred even for questions that mentioned the time and place, so we remain uncertain about the optimal resolution of this issue.

For the questions asked of post-partum women, differentiating between labour (stage 1) and delivery (stage 2) proved difficult, leading us to combine the original questions: ‘Were you allowed to have someone you wanted to stay with you during labour?’ and ‘Were you allowed to have someone you wanted to stay with you during delivery?’ into one: ‘When the baby was born, who was in the room?’ This amended question also avoided the respondent having to speculate about who could have been allowed with her, and instead tell us who was actually with her. It also emphasized presence in the same room, rather than asking more generally whether someone could be ‘with you’ since the cognitive interviews revealed that women could be denied birth companionship while family members waited in other rooms or outside the facility. Labour could be translated as ‘before the delivery’, ‘labour pain [prasav pida]’ or ‘before the baby came out’ and delivery could be translated to ‘while the baby was coming out’, ‘while you were pushing’ or ‘while in the delivery room’. However, after several cognitive interviews and after discussion with among researchers it became clear that attempting to divide the experience into two distinct parts would introduce excessive confusion for both respondents and the future quantitative enumerators. Pain is present in both phases, it was not always clear to women which locations within the facility were for which stage of labour (especially for women who arrived in the second stage of labour and immediately delivered), and the moment the baby emerged is only the final part of the second stage of labour.

### Question failure type 5: Accessing different cognitive domains

Questions were sometimes interpreted clearly by respondents, who gave confident answers, but were in fact accessing a different cognitive domain than was the interviewer and question developer’s intent (Table 4).

**Table 4:** Cognitive mismatch between respondent’s interpretation and question designer’s intent

Question	Question intent	Respondent interpretation	Resolution
Did the doctors, nurses or other health-care providers at the facility treat you with proper behaviour [acchaa vyavahaar]?	Was there good overall patient–provider rapport, was the patient treated with respect?	‘Proper behaviour’ is any behaviour that led to my and my baby’s survival.	Changed keyword from ‘proper behaviour’ to ‘maan sammaan’ [respect]
Would you recommend this facility to other women? / I would recommend this facility to other women.	What is the respondent’s overall impression of the quality of care provided?	To what extent do I feel confident enough to interact with and make recommendations to other women?	Removed the question
Would you return to this facility for future ANC/another delivery?/I would return to this facility for future ANC/another delivery.	What is the respondent’s overall impression of the quality of care provided?	Will I require future ANC/delivery care? Will I be able to afford and access alternative options in the future?	Removed the question

Hypothetical questions about potentially recommending the facility to other women or returning to the same place for future care failed to assess quality of care. Instead, respondents focused on their capacity to make recommendations and their hypothetical future care needs and options. Responses included: ‘No, how can I tell others what to do?’ and ‘I don’t speak to other women’. Several respondents who said they would go for ANC or delivery to the same place explained, upon probing, that they are unable to afford any other option so they will have to go back to the same facility: ‘We are poor, this is our only option’. One respondent (CT_ANC_03) said ‘no’ she would not go back, but selected this response because she had been sterilized after her child’s birth and had no plans to go for another delivery. When the interviewer probed about the hypothetical situation of a future birth she continued to adamantly assert that she had completed her childbearing and would not need to have another delivery.

### Question failure type 6: Failure to resonate with respondent worldview and realities

Some concepts failed to resonate with respondents’ worldviews and realities, particularly the concepts of being asked for consent, asking healthcare providers questions or having the reason for procedures/examinations explained, women being involved in decisions about care, women being able to choose from multiple positions for delivery and expectations of being greeted by providers.

Women conveyed a sense that they gave implicit consent by choosing to access healthcare and that social and gender norms limited their capacity to accept or refuse interventions. Women often said ‘yes’ they were asked for consent, although on discussion a number of examinations and procedures were done to them without anyone asking their permission. They expressed a conviction that the procedures done to them were implicitly allowed by nature of the power dynamic between healthcare provider and patient and because the procedures intended to ensure their wellbeing. The following replies exemplify this sense of acceptance and implied consent: ‘This is their work, checking [the vagina]’ (CT_PP_05); ‘I went there, so of course I wanted the doctor to do tests/procedures’ (CT_ANC_03); ‘It was hurting and it’s their work, so they will do it’; and ‘I knew the doctor would do an abdominal/vaginal check’. One respondent conveyed a sense that female providers did not require consent to touch female patients, saying: ‘They were ladies only’ (CT_PP_01).

Health providers explaining the reason for examinations, patients asking questions about their care, and patients being involved in decisions about their care were strange concepts to many women. They assumed that the reason for all examinations was to ensure the health of the mother and baby and did not expect to understand aspects of care, receive explanations or participate in decision-making:

*This is how it must be happening. I was not having any knowledge about all this… He is the doctor, he is working this much. He must be having knowledge about this… It was my first time, so I felt that it must be this way. This might be the process* (CT_PP_01).

The idea of being free to take a birth position of choice according to one’s comfort did not resonate with the respondents, who appeared unaware that there were any other positions beyond lying on one’s back for a facility delivery. One respondent (CT_PP_15) kept asking the researcher if the researcher meant to ask whether she was allowed to move around and change position after the delivery, because she could not understand being free to move from her back during delivery. Another respondent (CT_PP_09) explained that she had no preference or expectation:

I When the delivery was being done, when the child was being delivered, then the delivery occurred in a way/position you wanted or the way the nurse, and doctor were telling?

R Delivery was normal [e.g., vaginal].

I Fine, delivery was normal. […] So they made you to lie down or squat or…?

R They made me lie down.

I On your back?

R Yes.

I They made you lie down. Ok. Was that how you wanted the delivery to happen?

R I went for the first time so I was not aware how it would be.

I Ok, you are not aware. I am asking this because some do not want to lie down and deliver the child. Maybe we want to deliver by sitting down. So what was your wish?

R There was no wish from my side (CT_PP_09).

We attempted to resolve this issue by first asking the respondent which positions she took during the delivery, and reading options (lying on one’s back, standing, squatting, other) to essentially introduce the concept that being on one’s back was not the only possible position. We then asked whether the respondent chose the position(s) according to her comfort or whether she was made to take the position(s) by the staff.

Asking whether respondents were greeted by healthcare providers when they arrived at facilities, which aimed to assess whether positive patient–provider rapport was established, was difficult to adapt to this context. Literature from African settings suggests that healthcare providers failing to greet patients ‘is a rejection of social rules’ and that ‘women frequently expressed disapproval’ of not being greeted (Solnes Miltenburg *et al*., 2018, p. 102). However, in our study, respondents conveyed a sense that medical and social norms make warm or friendly greetings from providers to patients and from those with higher social status to those with lower social status abnormal and unexpected.

## Discussion

Our study identified severe cognitive failures across survey questions drawn from validated instruments used in other settings to measure RMC. Even questions on seemingly straightforward concepts, such as whether a woman had a birth companion or was allowed to eat and drink during labour, were initially framed in a manner that failed to access the intended cognitive domain. Study findings highlight the importance of cognitive interviews in the development of structured survey tools, particularly those involving complex or intangible concepts such as consent or personal private health information, and intended for use in marginalized populations. This article presents a typology of the following six common question failures to help organize and address cognitive interview results: (1) poor sequencing, length and sensitivity; (2) problematic response options; (3) inappropriate vocabulary and long sentences; (4) temporal and spatial confusion; (5) accessing different cognitive domains; and (6) failure to resonate with the respondent’s worldview and realities.

Some question failures were straightforward and relatively easy to address, such as sequencing questions in a manner that places the most sensitive questions towards the end, removing inappropriate or irrelevant components, changing or adding vocabulary words, and adding examples to improve comprehension.

Others were more challenging. Likert scales were found to be generally incomprehensible amongst pregnant and post-partum women in rural Madhya Pradesh and as such are not recommended for use in quantitative survey tools in this context. Efforts to use Likert scales were met with persistent challenges, despite trying a range of variations by colour, number of options, images, and wording. Respondents were very unfamiliar with the idea of graduated levels of agreement or disagreement with statements. Other researchers have similarly identified Likert scales as problematic for less-educated populations (Oyeyemi *et al*., 2016; Afulani *et al*., 2017; Nyongesa *et al*., 2017; Zongrone *et al*., 2018). Three-point answer options that assessed frequency (always, sometimes, never) or gradation for simple concepts (very clean, somewhat clean and somewhat dirty, very dirty) were more successful and appropriate for some questions.

Ensuring that respondents answer questions about the same time and place as the researcher intends was surprisingly difficult; respondents were frequently found to be thinking about different healthcare visits and time periods than the question intended.

Among the question failures identified, the most challenging to resolve were those which asked the respondent to consider hypothetical situations and those involving concepts from international literature which did not resonate with local worldviews or realities. In the context of RMC in rural India, hypothetical questions about future care and about recommendations to other women failed to access the intended cognitive domain (satisfaction with service quality). Instead, they assessed the respondents’ future childbearing intentions, ability to afford accessing other health facilities in the future and the quality of their relationships with other women. Many international RMC concepts resonated poorly or not at all among respondents in a health system characterized by unquestioned faith and adherence to medical knowledge, implicit consent, women’s low awareness of alternative protocols or options and pervasive gender norms and social hierarchy between patients and providers. These concepts included consent, patient rights to information privacy, patient involvement in decision-making over care, choosing a delivery position, providers explaining the reasons for and outcomes of clinical exams and expectations of being greeted by providers.

These issues of poor resonance are likely grounded in the normalization of low-quality care (Bowser and Hill, 2010), women’s low educational status (MoHFW, 2017), socialization that discourages women, particularly lower-status women, from questioning those in power (such as medical professionals) and gender norms that more broadly limit women’s control over major aspects of their lives (Gupta and Yesudian, 2006; MoHFW, 2016). In this context of limited female self-efficacy and decision-making power, women and providers have low inclination and expectation to engage in critical dialogue, information sharing and joint decision-making about maternity care. Our attempt to account for these issues involved removing some questions, such as the question on being involved in decisions about one’s care, and adjusting others (see Supplementary Table S1 for question-by-question details). Adjustments included changing the wording around ‘greeting’ to include multiple possible forms of establishing initial positive rapport; improving translation of key words; and adding examples of the types of procedures or examinations that may have been done to women to anchor questions about consent and explanations. However, the adjusted questions simply better-expressed concepts that ultimately had low resonance with many women’s understandings of quality maternity care. Additional research and critical discussion within the RMC community are required to identify more appropriate ways of assessing these constructs and to determine whether all global constructs can even be assessed in all populations, in the absence of broader improvements to women’s autonomy, self-efficacy and awareness of their rights.

Overall study findings led to the development of a refined structured survey for measuring RMC during pregnancy and childbirth in rural Hindi-speaking India. Despite the promise of these revised tools, alternative ways of measuring RMC should continue to be explored. One alternative may involve eliciting narrative description of women’s experiences to complete a structured quantitative survey, rather than asking quantitative questions directly. Our respondents were more comfortable narrating what happened to them than framing answers to quantitative questions. Moreover, while respondents frequently provided an immediate answer to the quantitative survey questions, subsequent qualitative descriptions often presented information that was entirely contradictory to what the initial quantitative response suggested, or revealed complete cognitive mismatch around the question’s intent. Other researchers have similarly found that women’s initial responses to questions about their treatment during childbirth vary drastically from their disclosures of disrespect and abuse after probing and rapport building (McMahon *et al*., 2014) or between quantitative and qualitative measures (Kambala *et al*., 2017). In addition to exploring alternative research methods to capture women’s perceptions of RMC, research seeking to fully document and understand women’s obstetric experiences must also triangulate using a range of other data inputs, including observation, qualitative work and companion interviews (Bazant and Huang, 2013). There are aspects of women’s experiences during maternity care that their companions may be more equipped to discuss, such as about demands for informal payments, which are often handled by male relatives. Observation by trained researchers has also identified higher rates of disrespect and abuse than are reported by women (Sando *et al*., 2016).

Efforts to conduct cognitive interviews in this context where challenged by difficulties interviewing women one-on-one without family members present. While efforts were made to conduct interviews in private, our researchers felt that it was culturally inappropriate to insist that curious family members leave the room. The result was that some mothers-in-law and husbands sat in on interviews and occasionally interjected. The qualitative approach taken in conducting and analysing these cognitive interviews differs from other cognitive testing of survey tools (Gibson *et al*., 2017). We were unable to calculate specific percentages for question failures or the number of respondents who understood or struggled with each question because the survey tools evolved iteratively over the course of the study. Moreover, in many cases, the interviews could not cover the entire survey tool because it was too long (common during cognitive interviews using the original tool) or because respondents had to attend to household and childcare duties.

## Conclusion

Cognitive interviews revealed a number of problems with RMC survey questions derived from validated tools in other contexts, which would not have been detected through typical pilot testing and which threatened the validity and reliability of the tool. Questions and response options were particularly poorly attuned to the realities and understandings of the most marginalized women, potentially ‘including’ them as respondents but excluding their experiences and opinions from being accurately captured.

Improving the measurement of women’s perspectives and experiences must be a priority in the RMC field and beyond. Poorly developed survey tools misrepresent women’s experiences and fail to assess important but difficult-to-measure domains. In addition to rigorous cognitive testing for new survey questions, new translations and tools developed in different settings, we must also identify better ways to measure the aspects of women’s experiences that cannot be captured through traditional quantitative surveys. Only then will research understand women’s experiences and contribute to improving services.
