# Supplementary material for: Yes, no, maybe so: the importance of cognitive interviewing to enhance structured surveys on respectful maternity care in northern India
Source: Health Policy Plan. 2019 Oct 31:10.1093/heapol/czz141. doi: 10.1093/heapol/czz141 (PMC7053388; doi:10.1093/heapol/czz141)
Supplement: Supplementary file 1 [file HPP-2019-HEAPOL-CZZ141-S1.docx]

**Figure 1. Likert scales developed for cognitive testing**

| 1 | 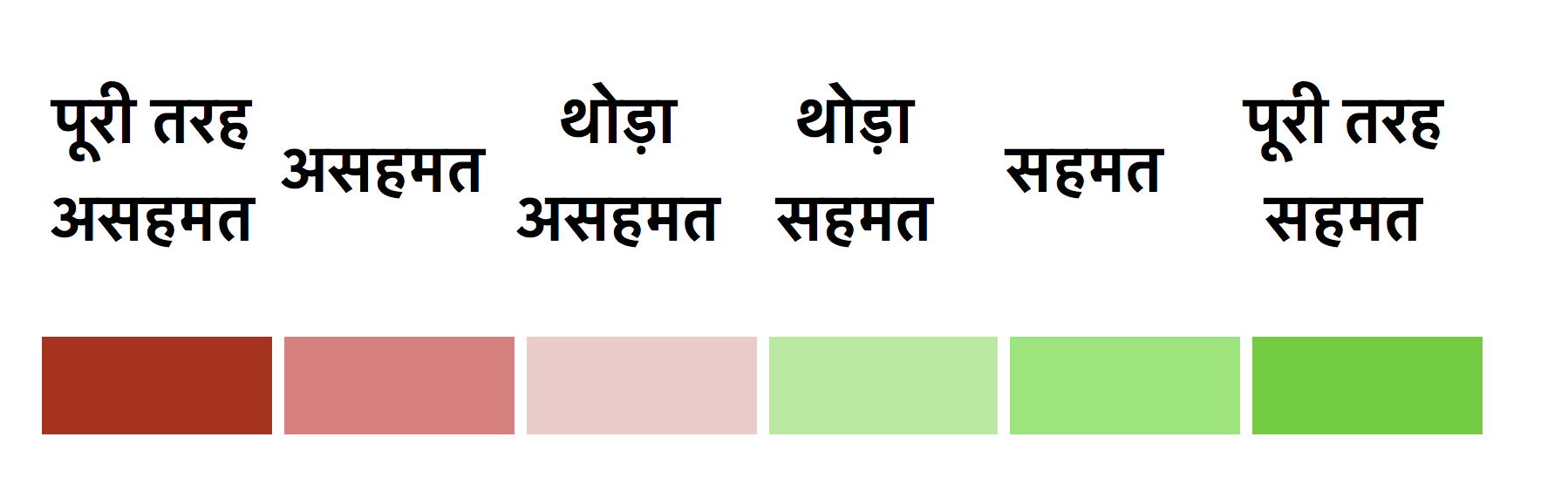  Translation (not included in scale):  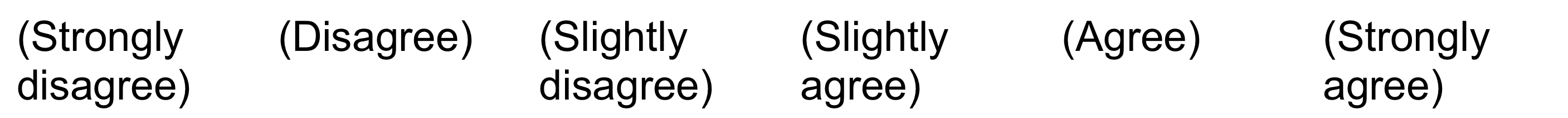 | 2 | 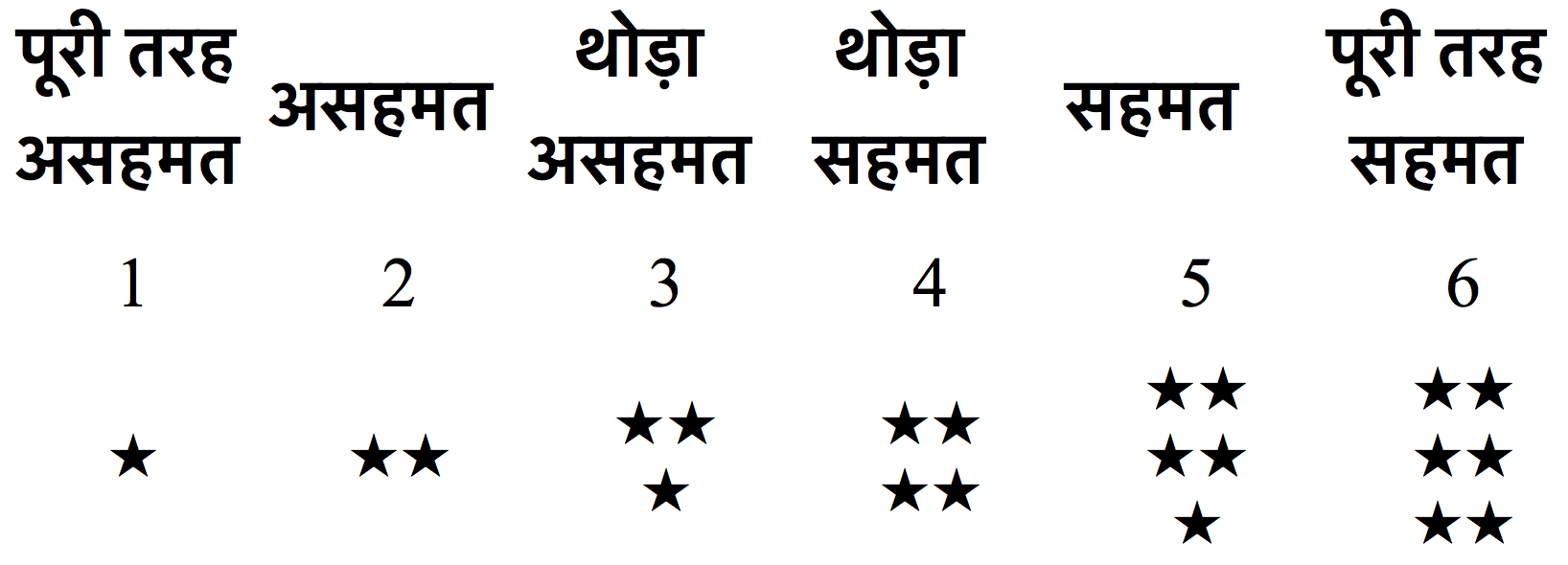 |
| --- | --- | --- | --- |
| 3 | 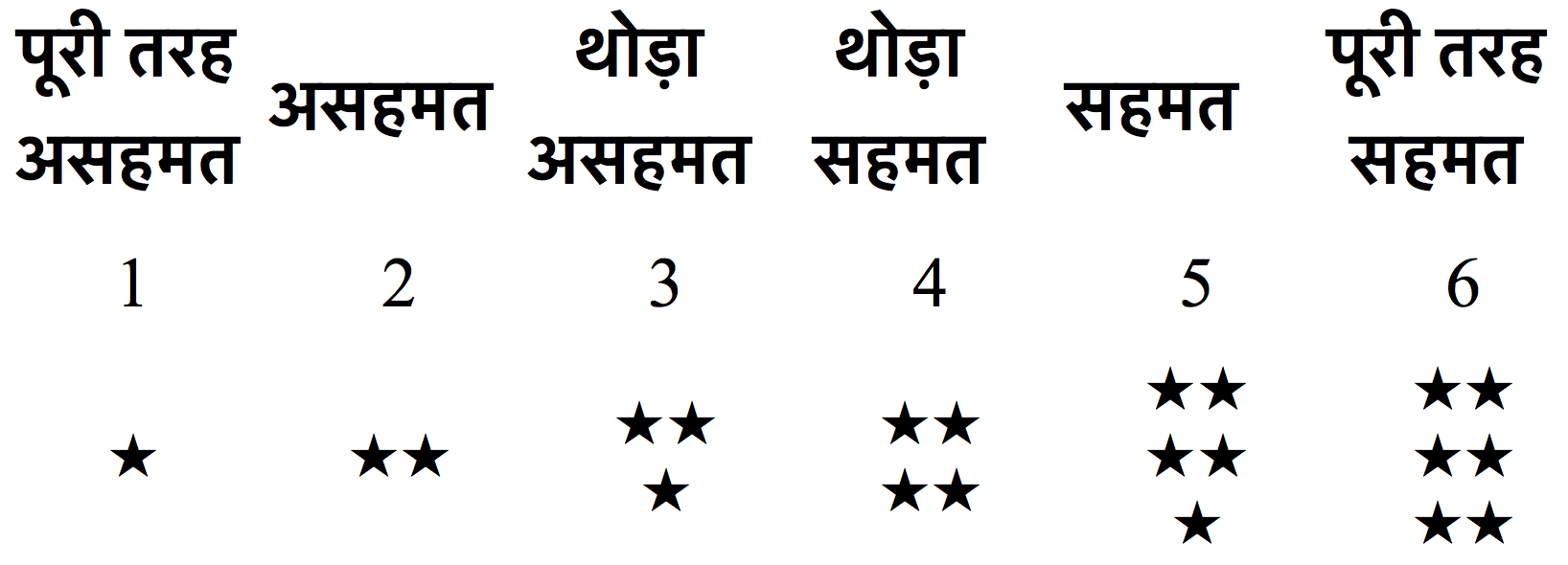 | 4 | 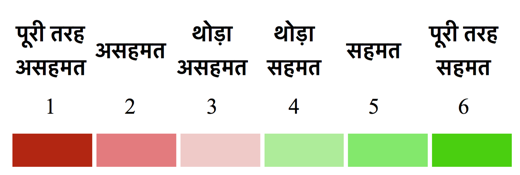 |
| 5 | 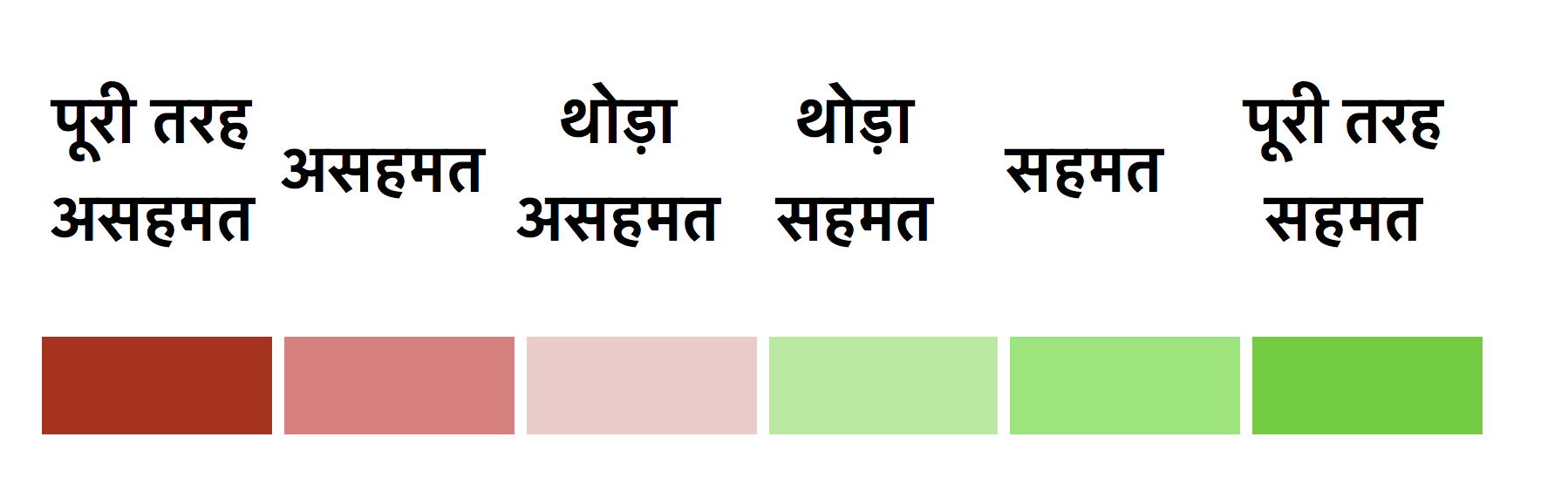 | 6 | 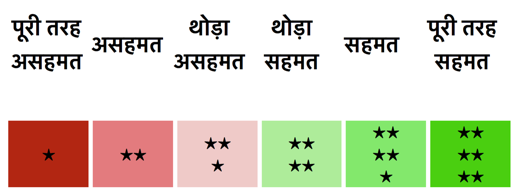 |
| 7 | 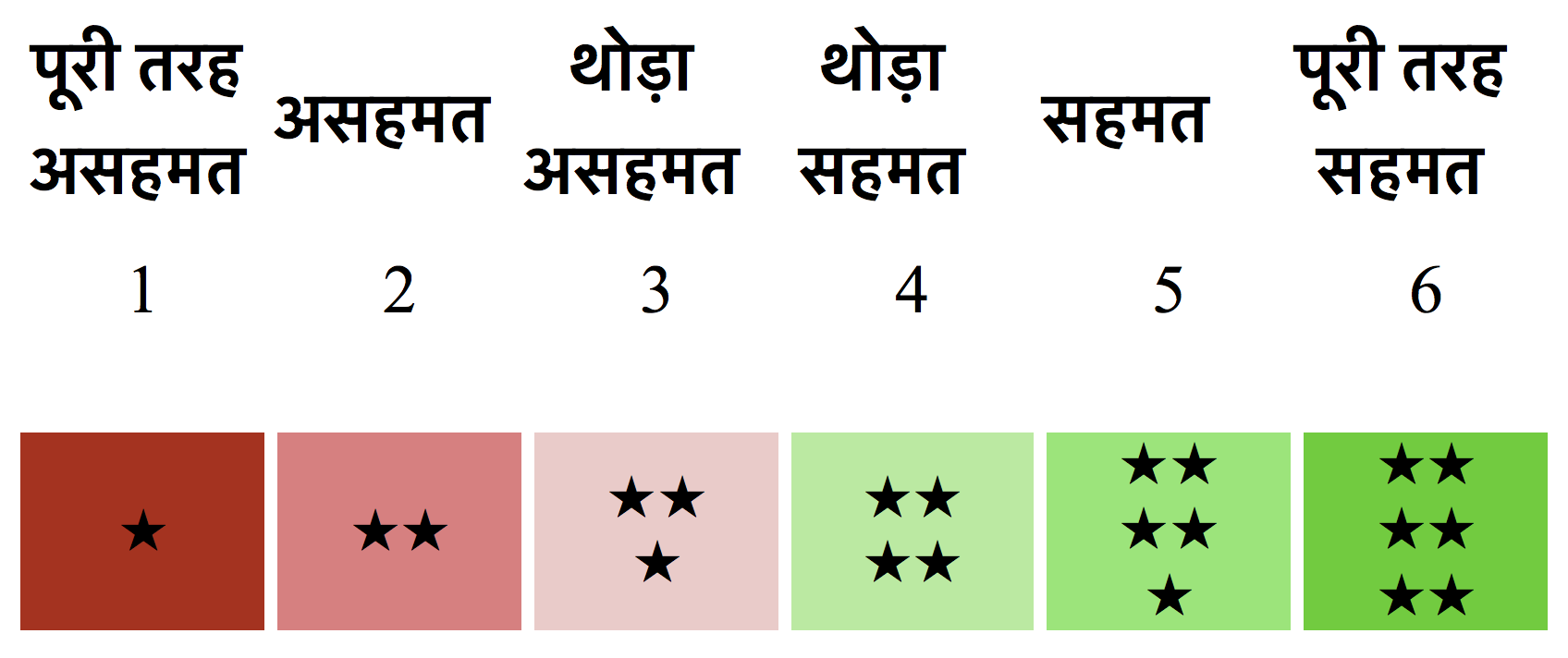 | 8 | 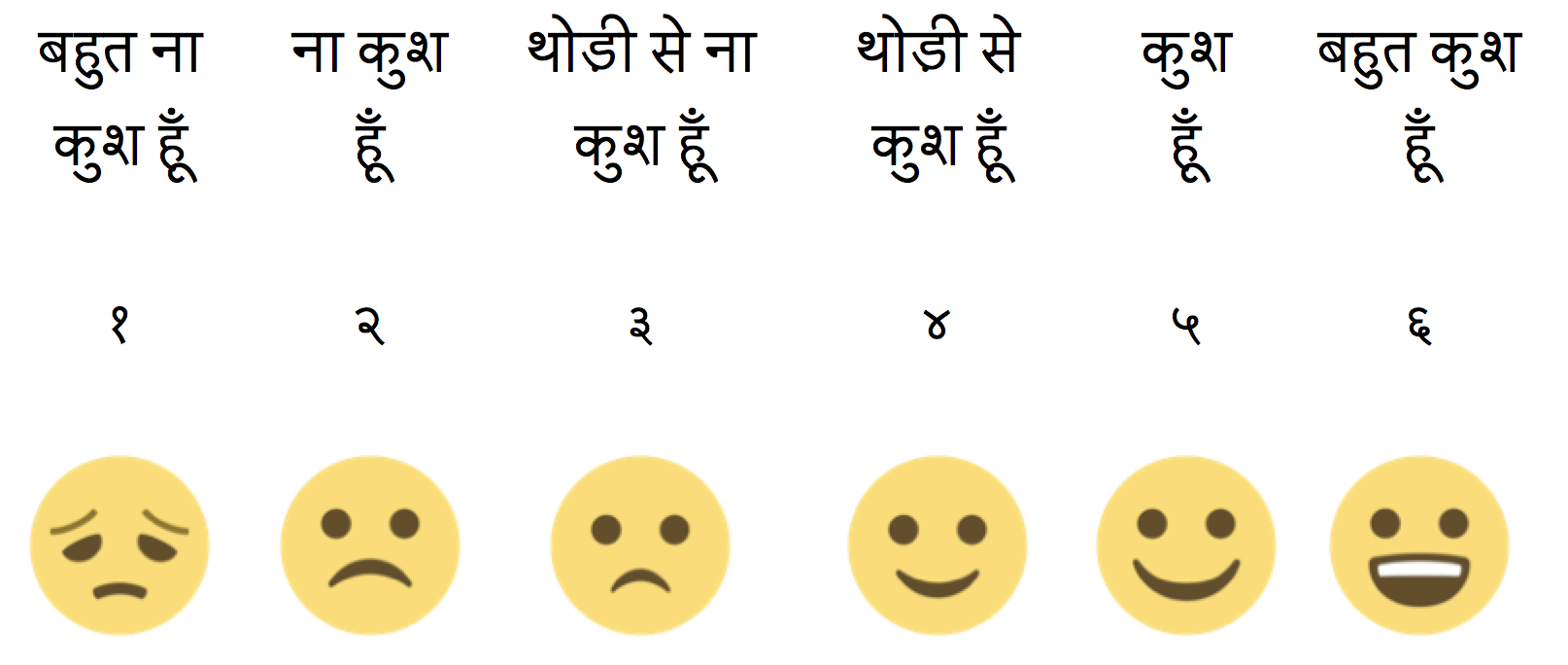  Translation (not included in scale):  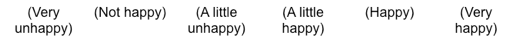 |
| 9 | 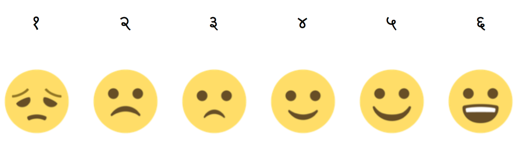 | 10 | 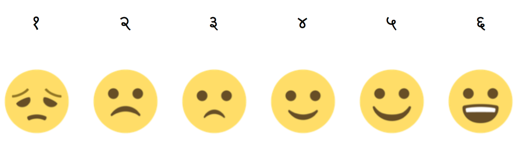 |
| 11 | 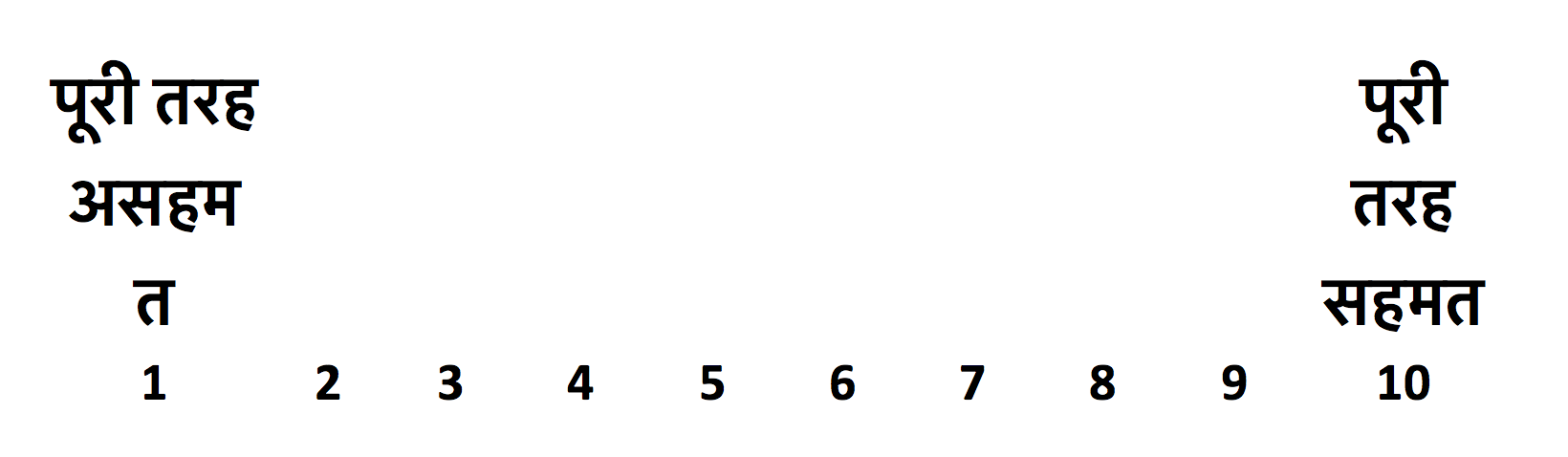 | 12 | 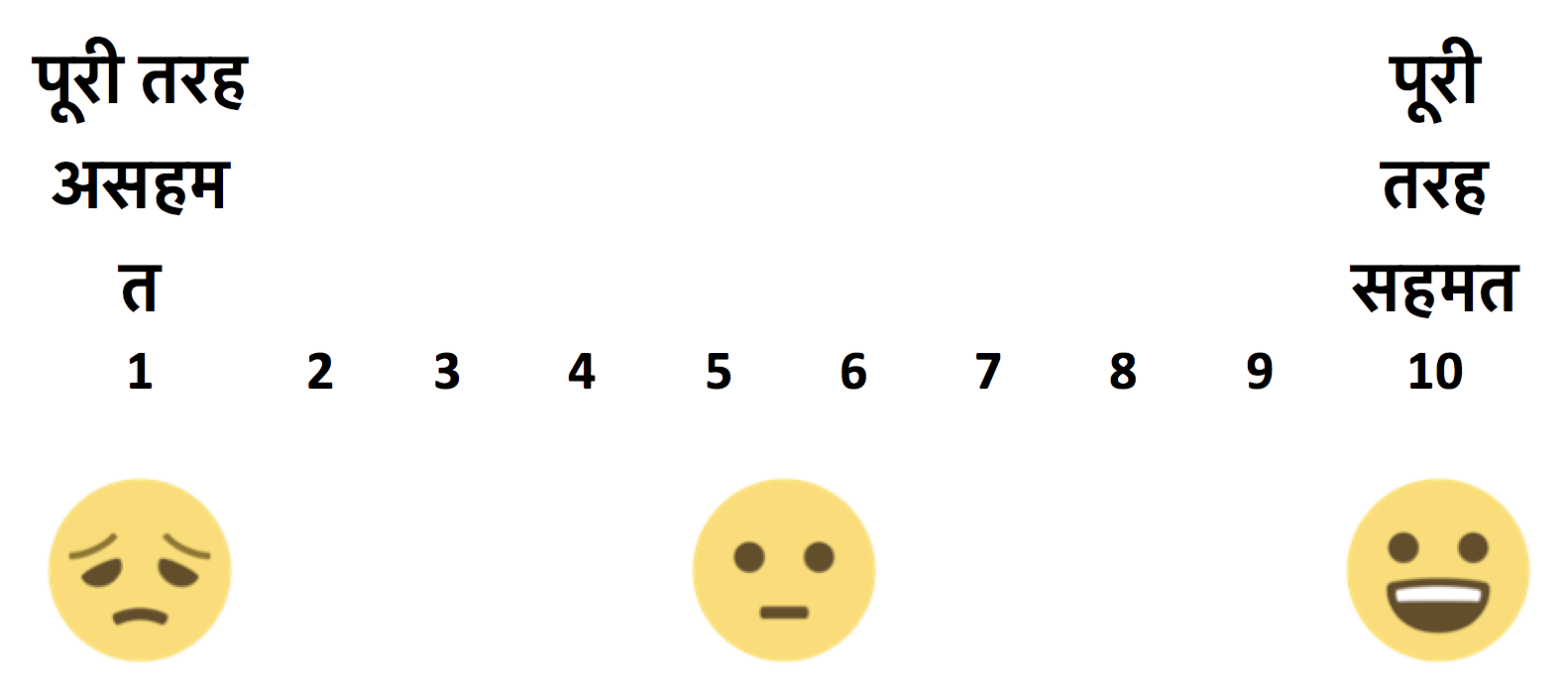 |
| 13 | 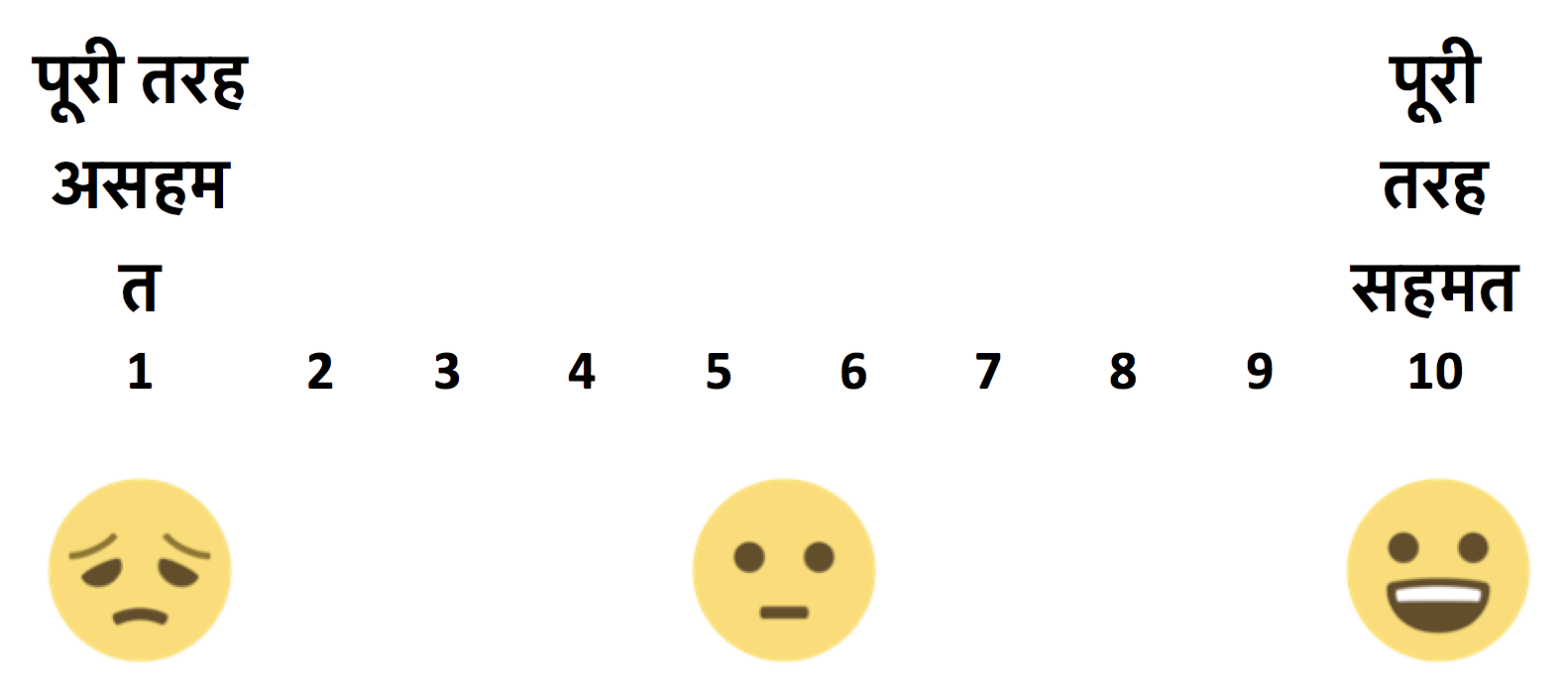 | 14 | 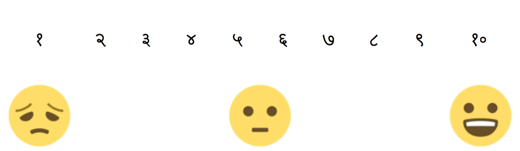 |
