# Supplementary material for: Yes, no, maybe so: the importance of cognitive interviewing to enhance structured surveys on respectful maternity care in northern India
Source: Health Policy Plan. 2019 Oct 31:10.1093/heapol/czz141. doi: 10.1093/heapol/czz141 (PMC7053388; doi:10.1093/heapol/czz141)
Supplement: Supplementary file 2 [file HPP-2019-HEAPOL-CZZ141-S2.docx]

**Figure 2. Example section of the cognitive interview guide**

| Question 1. “When you went for delivery to the health facility did the doctors, nurses, or other health care providers introduce themselves to you when they first came to see you?”  Response options: yes, no, don’t know, no response  Cognitive probes:   - What happened when you first met the doctors, nurses, or other health care providers? - What did they say to you? - Can you tell me about how they spoke to you when you arrived? What kinds of things did they say? How did they sound – speaking with love/nicely or harshly? - What does “introduction” [parichay] mean to you? What other word could we use? - Did you find this question easy to understand/answer? Was it easy to remember what happened? |
| --- |
