# Supplementary material for: Yes, no, maybe so: the importance of cognitive interviewing to enhance structured surveys on respectful maternity care in northern India
Source: Health Policy Plan. 2019 Oct 31:10.1093/heapol/czz141. doi: 10.1093/heapol/czz141 (PMC7053388; doi:10.1093/heapol/czz141)
Supplement: Supplementary file 3 [file HPP-2019-HEAPOL-CZZ141-S3.docx]

**Figure 3. Examples of Likert response option failures**

| *Example 1. Respondent selects smiley face scale response based on her emotional state at the time, rather than the issue in question*  For the question: “The doctors, nurses or other health care providers at the facility did everything they could to help control my pain” a respondent pointed to the saddest face [left-most] in the Likert scale below:  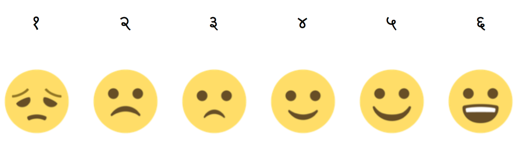  On probing she explained that the staff were supportive and spoke nicely to her, but that since she was in pain her face was “like that” so she selected the saddest face.  *Example 2. Respondent does not engage with Likert scale and instead provides a dichotomous response*  Many respondents avoided engaging with the Likert scales, instead continuing to repeat their response in terms of a dichotomous yes/no, agree/disagree, happened/did not happen, etc.. For example, in response to the statement about procedures being explained before they were conducted, a respondent (CT_PP_05) repeated “they didn't explain” several times while the interviewer attempted unsuccessfully to get her to convert this response into a Likert option.  *Example 3. Respondent selects a response based on how she felt about the information or situation referred to by the statement, rather than her level of agreement with the statement*  In some cases, the respondent selected a Likert scale response but was indicating how she felt about the information or situation, rather than her level of agreement with the statement. For example, when responding to the statement “The results of examinations were explained to me” a respondent (CT_PP_05) replied that she agreed “a little bit,” which the researchers initially thought could be transferred to the Likert scale as “somewhat agree.” However, she was not reporting that only some results were explained to her. Instead, she selected “a little bit” because the result of the examination was negative, as conveyed in the conversation below.  R: They told me. I mean, they told me. There was an issue. Cord was stuck on the baby's neck, and they told me.  I: They told you that. But do you completely agree, or a little bit? R: A little bit.  I: Why?  R: It was in the baby's neck. I wasn't happy. (CT_PP_05) |
| --- |
