# Supplementary material for: Yes, no, maybe so: the importance of cognitive interviewing to enhance structured surveys on respectful maternity care in northern India
Source: Health Policy Plan. 2019 Oct 31:10.1093/heapol/czz141. doi: 10.1093/heapol/czz141 (PMC7053388; doi:10.1093/heapol/czz141)
Supplement: Supplementary file 4 [file HPP-2019-HEAPOL-CZZ141-S4.docx]

**Table 1. Respondent sample**

|  | Original tool | Revised tool 1 | Revised tool 2 | *Total* |
| --- | --- | --- | --- | --- |
| Postpartum women | 8 | 4 | 3 | *15* |
| Pregnant women | 4 | 1 | 1 | *6* |
| *Total* | *12* | *5* | *4* | *21* |
