# Supplementary material for: Yes, no, maybe so: the importance of cognitive interviewing to enhance structured surveys on respectful maternity care in northern India
Source: Health Policy Plan. 2019 Oct 31:10.1093/heapol/czz141. doi: 10.1093/heapol/czz141 (PMC7053388; doi:10.1093/heapol/czz141)
Supplement: Supplementary file 5 [file HPP-2019-HEAPOL-CZZ141-S5.docx]

**Table 2. Typology of RMC survey question failures, identified through cognitive interviews**

| Question failure type | Explanation | Example (see text and S1 for more examples) |
| --- | --- | --- |
| Issues with sequencing, length and sensitivity | Question order does not flow well, sensitive questions come too early (before there has been time to establish adequate rapport), respondents find the tool long and/or repetitive. | Questions about physical abuse during pregnancy were initially placed early in the survey before sufficient rapport was established, which made respondents uncomfortable and unlikely to disclose negative experiences. |
| Problematic response options | Response options fail to capture frequent replies, are inappropriate, or are confusing to respondents. | Likert scales and the concept of graduations of agreement or disagreement along a spectrum were incomprehensible to most respondents and failed to capture meaningful responses. |
| Inappropriate vocabulary and long sentences | Key vocabulary terms not locally understood; long sentences and sentences with multiple components are difficult for respondents to follow. | The initial translations of key words such as delivery, health centre, physically harmed, sterilization, insurance, vaginal, and many others were not understood. |
| Temporal and spatial confusion | Mismatch between the time and location that the survey question was seeking to assess and the time and location that respondents considered. | When a respondent was asked whether she was allowed to drink liquids or eat any food while in labor she replied “yes”. Upon probing she told us about the nutrition advice she received from the health facility staff throughout her pregnancy. |
| Accessing different cognitive domains | Question accesses a different cognitive domain than was the interviewer and question developer’s intent. | A respondent replied that she would return to the same place of delivery in the future; but on probing we found that she was thinking about her inability to afford health care elsewhere (in a private facility) rather than her satisfaction with the services she received. |
| Failure to resonate with the respondent’s worldview and reality | Question asks about a domain of global importance that does not align with local assessments of respectful care. | Respondents expected health care providers to use their knowledge and experience to make decisions about the best course of action for the woman and her baby; women did not understand the idea of being involved in decisions about their care. |
