# Supplementary material for: Yes, no, maybe so: the importance of cognitive interviewing to enhance structured surveys on respectful maternity care in northern India
Source: Health Policy Plan. 2019 Oct 31:10.1093/heapol/czz141. doi: 10.1093/heapol/czz141 (PMC7053388; doi:10.1093/heapol/czz141)
Supplement: Supplementary file 6 [file HPP-2019-HEAPOL-CZZ141-S6.docx]

**Table 3. Vocabulary issues and potential resolutions**

| Issue | Original | Improved alternative |
| --- | --- | --- |
| Anglicised words more familiar than academic / sanskritized Hindi words | Swasthya kendra [health centre] | Aaspital [hospital] |
|  | Prasav [delivery] | Dilivri [delivery] |
|  | Sahamati [consent] / anumahati [permission] | Parmishin [permission] |
| Academic / Sanskritized words unknown, simple or common Hindi words more widely used | Sharirik roop se [bodily/physically] | Can avoid the word by asking if anyone hurt you – respondents assume we mean physically hurt |
|  | Udaaharan ke liye [for example] | Jaise [like] / matlab [meaning] |
|  | Parichay [introduction] | Aapse jaan-pahachaan [your acquaintance]/naam bataayaa [told you their name]/haalchaal poochi [asked your wellbeing] |
| Different words understood by different women, thus multiple options provided | Guptang [genital] | Yoni [vagina]/ batcha hone wali jagah [baby place] |
|  | Ukadoon baithana [squat] | Ghutane mod ke [knee bent]/ ukadoon baithana [squat]/ toilet letareen karate samay jaise baithe hai [sitting on toilet] |
| Key word not understood. The use of examples aided comprehension. | During your hospital stay, did health providers ever discuss your personal **private [neejith]** health information in a way that others could hear? | Did health providers ever discuss your personal **private [neejith]** health information in a way that others could hear? For example, healthcare providers could say the results of your lab reports loudly, so that others might hear it. |
|  | Did the doctors, nurses or other staff at the facility ask your **consent [sahamati]** before doing your examinations? | Before doing the vaginal exam or any other exam, did the health workers **ask you if they could do it / ask permission [parmishan]**? |
