# Supplementary material for: Yes, no, maybe so: the importance of cognitive interviewing to enhance structured surveys on respectful maternity care in northern India
Source: Health Policy Plan. 2019 Oct 31:10.1093/heapol/czz141. doi: 10.1093/heapol/czz141 (PMC7053388; doi:10.1093/heapol/czz141)
Supplement: Supplementary file 7 [file HPP-2019-HEAPOL-CZZ141-S7.docx]

**Table 4. Cognitive mismatch between respondent’s interpretation and question designer’s intent**

| **Question** | **Question intent** | **Respondent interpretation** | **Resolution** |
| --- | --- | --- | --- |
| Did the doctors, nurses or other health care providers at the facility treat you with proper behaviour [acchaa vyavahaar]? | Was there good overall patient-provider rapport, was the patient treated with respect? | “Proper behaviour” is any behaviour that led to my and my baby’s survival. | Changed key word from “proper behaviour” to “maan sammaan” [respect] |
| Would you recommend this facility to other women? / I would recommend this facility to other women. | What is the respondent’s overall impression of the quality of care provided? | To what extent do I feel confident enough to interact with and make recommendations to other women. | Removed the question |
| Would you return to this facility for future ANC/another delivery? / I would return to this facility for future ANC/another delivery. | What is the respondent’s overall impression of the quality of care provided? | Will I require future ANC/delivery care?  I will be able to afford and access alternative options in the future? | Removed the question |
