# Supplementary material for: Yes, no, maybe so: the importance of cognitive interviewing to enhance structured surveys on respectful maternity care in northern India
Source: Health Policy Plan. 2019 Oct 31:10.1093/heapol/czz141. doi: 10.1093/heapol/czz141 (PMC7053388; doi:10.1093/heapol/czz141)
Supplement: Supplementary file 8 [file HPP-2019-HEAPOL-CZZ141-S8.docx]

**Supplementary table S1. Original and revised RMC survey questions with notes on revisions made**

| **No** | **Original question** | **Original response option** | **No** | **Revised question** | **Revised response option** | **Notes** |
| --- | --- | --- | --- | --- | --- | --- |
| 1 | During your time in the health facility did the doctors, nurses, or other health care providers introduce themselves to you when they first came to see you?  जब आप स्वास्थ्य केंद्र पर पहली बार डॉक्टर, नर्स या किसी अन्य स्वास्थ्यकर्मी से मिली तो क्या उन्होनें आपको अपना परिचय दिया? | Yes हाँ; No नहीं; Don’t know पता नहीं | 1 | I would like to ask you about the health workers, meaning doctors, nurses, etc. When you went for delivery to the health facility did any health workers greet you, like, did they tell their name or ask about your wellbeing or speak to you nicely?  में आपसे स्वस्थ्यकर्मियों के बारे  में कुछ सवाल पूछना चाहती हूँ।स्वास्थ्यकर्मी यानी डॉक्टर, नर्स, अन्य कर्मचारी। जब आप डिलेवरी के लिए अस्पताल गयी थी तब क्या उन्होंने आपसे जान-पहचान की, जैसे की अपना नाम बताया या आपका हालचाल पूछा या आपसे अच्छे से बातचीत कि? | Yes हाँ; No नहीं; Don’t know पता नहीं; No response / refused to answer जवाब नहीं दिया/ जवाब देने से  मनाकिया | - Garbhavastya [delivery] changed to dilivri [delivery] - Swasthya kendra [health centre] changed to aaspital [hospital] - Health worker explained in separate sentence as “doctor, nurse, or any other worker” - Parichay [introduction] changed to jaan-pahachaan [your acquaintance] - Added “like, did they tell their name or ask you about your wellbeing or speak to you nicely?” |
| 2 | At any point during your stay for this delivery were you physically harmed by any of the health care workers? For example, physical abuse might include being hit, pinched, pulled or slapped  प्रसव के लिए स्वास्थ्य केंद्र में रुकने के दौरान क्या किसी भी स्वास्थकर्मी के दुवरा आपको शारीरीक रुप से तकलीफ पहुचाई गयी? जैसे की पीटना, चिकोटी काटना, जोर से खींचना या थप्पड़ मारना। | Yes हाँ; No नहीं; Don’t know पता नहीं; No response/Refusal to answer उत्तर नहीं दिया/ उत्तर देने से इंकार किया | NA | NA | NA | - Original question #2 and #3 combined into revised question #29 (below) - New #29 asks all women about physical mistreatment and reads all women the response options because many may say “no” to a broad question (original #2) and then miss hearing the specific types of mistreatment that could help them identify and remember mistreatment |
| 3 | (SKIP 3 IF IN 2 WOMAN SAID: NO, DON’T KNOW, NO RESPONSE) What exactly happened? (DO NOT READ, Circle all that apply, prompt for any more)  (प्रश्न 3 को छोड़ करें आगे बढ़े, यदि उत्तरदाता ने 2 का उत्तर: नहीं, नहीं पता/ उत्तर नहीं दिया)  किस तरह का व्यवहार हुआ था आपके साथ?  (वोकल्पों को ना पढ़े, लागू होने वाली उत्तरों को गेर दें। | Kicked लात मारी; Pinched चिकोटी काटी;  Slapped थप्पड़ मारा; Pushed धक्का दिया; Beaten पीटा; Raped बलात्कार किया;  Tied to the delivery bed/delivery coach प्रसव वाले बिस्तर से बाँध दिया;  Other (SPECIFY) अन्य (उल्लेखकरें) | 29 | Now I would like to ask you about whether any of the health workers hurt or harmed you during your stay for this delivery. Did any healthcare provider ___?  [READ RESPONSES ONE BY ONE AND CIRCLE ALL THAT APPLY]  अब मैं आप से जानना चाहूंगी कि डिलीवरी के लिए अस्पताल में रुकने के दौरान, क्या किसी भी स्वास्थ्यकर्मी ने आप कोचोट या नुकसान पहुचाया था? आपको अस्पताल के किसी भी स्वास्थ्यकर्मी ने क्या _________? [सारे विकल्प पढ़कर बताएं और लागू होने वाले विकल्पों को गोले में घेरदें।] | Intentionally rough / painful exam (like vaginal exam) or treatment (like injection) जान बूझकर  कठोर / दर्दनाक तरीके से जांच किया जैसे, योनि की जाँच या दर्दनाक तरीके से टीका लगाया था;  Forcefully held / tied जोर से पकड़ा था/ बांधा था;  Kicked लात मारी थी; Pinched चिकोटी काटी थी;  Slapped थप्पड़ मारा था; Pushed धक्का दिया था; Beaten पीटा था;  Other (specify) अन्य (उल्लेखकरें);  No physical mistreatment reported कोई शारीरिक दुर्व्यवहार नहीं हुआ;  Don’t know नहीं पता; No response/Refused to answer उत्तर नहीं दिया/ देने से इंकार किया | - “Physically harmed” [sharirk roop se] in original #2 was not well understood, changed to “hurt” [chot] and harm [nuksaan] with physically implied - Interviewer now directly asks all respondents “Were you kicked?” “Were you pinched” and so on, to there is no confusion about what counts and does not not count as “hurt” or “physical harm” - “Intentionally rough / painful exam (like vaginal exam) or treatment (like injection)” added since this was the most common type of physical abuse mentioned in our interviews; “intentionally” added since some examinations and treatments can be painful without intent - Question moved towards end of survey |
| 4 | (SKIP 4 IF IN 2 WOMAN SAID: NO, DON’T KNOW, NO RESPONSE) Do you think that the poor treatment you described was influenced by any of the following? Please tell us the most important (PLEASE READ OUT THE OPTIONS AND CIRCLE ALL THAT APPLY)  (प्रश्न 4 को छोड़ करें आगे बढ़े, यदि उत्तरदाता ने 2 का उत्तर: नहीं, नहीं पता/ उत्तर नहीं दिया)  क्या आपको लगता है आपके साथ हुए बुरे व्यवहार की वजह इन बातों में से कोई कारण था, जो आपको सबसे जरुरी लगे हमें वो बताएं। (सारे विकल्प पढ़कर बताएं और लागु होने वाले विकल्पों को गोले में घेर दें।) | Your social class आपका सामाजिक तबका;  Lack of insurance बीमा न होना;  Your caste आपकी जाति;  Your sex आपका महिला होना;  Your language आपकी भाषा;  Your religion आपका धर्म;  Your political beliefs or other beliefs आपके राजनीतिक या कोई और विश्वास;  Your health status आका स्वास्थ्य;  Lack of money or wealth पैसे या संपत्ति की कमी;  Your age आपकी उम्र; Other (specify) अन्य (उल्लेख करें);  Not Applicable लागू नहीं; Don’t know पता नहीं; No Response/Refusal to answer उत्तर नहीं दिया/ देने से इंकार किया | 30 | [SKIP 30 IF IN 29 WOMAN RESPONDED: NO PHYSCIAL MISTREATMENT OR DON’T KNOW]  Do you think that the poor treatment you described was influenced by any of the following?  [PLEASE READ OUT THE OPTIONS AND CIRCLE ALL THAT APPLY]  [यदि 29 का जवाब किसी प्रकार की शारीरीक चोट नहीं पहुचाई गई या  जवाब नहीं पता हो तो 30 के प्रश्न को छोड़ आगे बढ़ें।]  आपको क्या लगता है कि आपके प्रति ख़राब व्यव्हार या बर्ताव का कारण इनमें से कोई था? [सारे विकल्प पढ़ कर बताएं और लागु होने वाले विकल्पों को गोले में घेर दें] | Lack of money or wealthपैसे या संपत्ति की कमी;  Your caste आपकी जाति;  Your language आप की भाषा;  Your religion आपका धर्म;  Your political beliefs or other beliefsआप के राजनीतिक या कोई और विश्वास / धारणा;  Your health status आपकी सेहत;  Your ageआप की उम्र; Other (specify) अन्य (उल्लेखकरें);  Not Applicable लागू नहीं;  Don’t know नहीं पता;  No Response / Refused to answer उत्तर नहीं दिया/ देने से इंकार किया | - Removed social class, insurance and sex as response options: social class and lack of money and wealth are very similar, insurance is not understood at all and sex (“that you are a woman”) is confusing to respondents since the entire process of delivering a baby only occurs because they are female - Moved “lack of money or wealth” up to the top of the list because it strongly overlaps with “social class” |
| 5 | At any point during your stay for this delivery did any health care provider talk or behave rudely? प्रसव के लिए स्वास्थ केंद्र में रुकने के दौरान क्या किसी भी स्वास्थकर्मी ने आपसे बुरे तरीके से बातचीत या किसी प्रकार का बुरा व्यवहार किया? | Yes हाँ; No नहीं; Don’t know पता नहीं; No Response/ Refusal to answer उत्तर नहीं दिया/ उत्तर देने से इंकार किया | NA | NA | NA | - Original question #5 and #6 combined into revised question #27 - New #27 asks all women about verbal mistreatment and reads all women the response options because many may say “no” to a broad question (original #5) and then miss hearing the specific types of mistreatment that could help them identify and remember mistreatment |
| 6 | (SKIP 6 IF IN 5 WOMAN SAID: NO, DON’T KNOW, NO RESPONSE) What exactly happened? (READ RESPONSE AND CIRCLE ALL THAT APPLY)  (प्रश्न 6 को छोड़ करें आगे बढ़े, यदि उत्तरदाता ने 5 का उत्तर: नहीं, नहीं पता/ उत्तर नहीं दिया)  आपके साथ किस तरह का व्यवहार हुआ था? (पढ़कर ना बताएं, लागू होने वाले सारे जवाबों को गोले में घेर दें।) | Shouted चिल्लाया; Scolded डाटा; Threatened to withhold services स्वास्थ्य सेवाएंन रोक देने की धमकी दी; Threatened with going to operation theatre ऑपरेशन वाले कमरे में ले जाने की धमकी दी;  Called by insulting name अपमानजनक नाम से पुकारा; Laughed at or scorned मजाक उड़ाया;  Looked in a derogratory way गन्दी नज़र से देखा; Other (Specify) अन्य (उल्लेख करें);  Don’t know नहीं पता | 27 | Now I would like to ask you about whether any health provider talked or behaved rudely. Did any healthcare provider____?  [READ RESPONSE AND CIRCLE ALL THAT APPLY]  अब मैं आपसे जानना चाहूंगी कि क्या किसी स्वास्थ्यकर्मी ने आपसे कोई बुरा व्यव्हार या बर्ताव किया या बुरे ढंग से बात की? आपको अस्पताल के किसी स्वास्थ्यकर्मी ने क्या _________?[सारे विकल्प पढ़कर बताएं और लागू होने वाले विकल्पों को गोले में घेर दें] | Shouted चिल्लाया था; Scolded डांटा था; Threatened to withhold services स्वास्थ्य सेवाएं रोक देने की धमकी दी; Threatened with caesarean section ऑपरेशन करने की/पेटकाट के बच्चा निकालने की धमकी दी; Called by insulting name अपमान जनक नाम से पुकारा;  Laughed at or scorned मजाक उड़ाया; Looked in a derogatory way गन्दी नज़र से देखा;  Other (specify) अन्य (उल्लेख करें);  No verbal mistreatment reported कोई मौखिक दुर्व्यवहार नहीं हुआ;  Don’t know नहीं पता; No response/Refused to answer जवाब नहीं दिया/जवाब देने मना किया | - Reivsed question asks all women about potential types of verbal mistreatment, with no priorscreening question (original #5) which would have introduced a skip if response was negative - Question moved towards end of survey |
| 7 | (SKIP 7 IF IN 5 WOMAN SAID: NO, DON’T KNOW, NO RESPONSE) Do you think that the poor treatment you described was influenced by any of the following? Please tell us the most important (PLEASE READ OUT THE OPTIONS AND CIRCLE ALL THAT APPLY)  (प्रश्न 7 को छोड़ करें आगे बढ़े, यदि उत्तरदाता ने 5 का उत्तर: नहीं, नहीं पता/ उत्तर नहीं दिया)  क्या आपको लगता है आपके द्वारा बताएं गये  बुरे व्यवहार की वजह इन बातों में से कोई था, जो आपको सबसे जरुरी लगा हमें वो बताएं। (सारे विकल्प पढ़कर बताएं और लागु होनेवाले विकल्पों को गोले में घेर दें।) | Your social class आपका सामाजिक तबका;  Lack of insurance बीमा का न होना; Your caste आपकी जाति;  Your sex आपका महिला होना; Your language आपकी भाषा;  Your religion आपका धर्म;  Your political beliefs or other beliefs आपके राजनीतिक या कोई और विश्वास;  Your health status  आपका स्वास्थ्य; Lack of money or wealth पैसे या संपत्ति की कमी;  Age आपकी उम्र; Other (specify)  अन्य (उल्लेखकरें);  Not Applicable लागू नहीं;  Don’t know नहीं पता; No Response/Refusal to answer उत्तर नहीं दिया/ देने से इंकार किया | 28 | [SKIP28 IF IN27WOMAN SAID: NOVERBAL MISTREATMENT OR DON’T KNOW]  Do you think that the poor treatment you described was influenced by any of the following?  [PLEASE READ OUT THE OPTIONS AND CIRCLE ALL THAT APPLY]  [प्रश्न संख्या 27 का जवाब अभद्र व्यवहार का ना होना हो या  नहीं पता हो तो, प्रश्न 28 को छोड़ आगे बढ़ें।]  क्या आपको लगता है कि आपके प्रति ख़राब व्यवहार या बर्ताव /रवैया का कारण इनमें से कोई था? [सारे विकल्प पढ़कर बताएं और लागू होने वाले विकल्पों को गोले में घेर दें] | Lack of money or wealth पैसे या संपत्ति की कमी;  Your caste आपकी जाति;  Your language आपकी भाषा;  Your religion आपका धर्म;  Your political beliefs or other beliefs आपके राजनीतिक या कोई और विश्वास;  Your health status आपकी सेहत;  Age आपकी उम्र;  Other (specify) अन्य (उल्लेखकरें);  Not Applicable लागू नहीं;  Don’t know पता नहीं; No Response/Refused to answer उत्तर नहीं दिया/ देने से इंकार किया | - Removed social class, insurance and sex as response options - Moved “lack of money or wealth” up to the top of the list because it strongly overlaps with “social class” |
| 8 | Did the doctors and nurses explain to you why they were doing examinations or procedures on you?  क्या आपको डाक्टर या नर्स ने बताया/समझाया कि वो आपकी जांच क्यों कर रहे हैं? | Yes हाँ; No नहीं; Don’t know नहीं पता | 2 | Did the health workers tell you why they were doing examinations / checkups to you? Like, healthcare providers could explain why they are doing an abdominal examination before doing it? Did they: always tell, sometimes tell or never tell?  क्या स्वास्थ्यकर्मी ने कोई जाँच / चेकअप करने से पहले क्या आपको बताया था की वो वह जाँच / चेकअप क्यूँ कर रहे है?  जैसेके, पेट के जाँच / चेकअप करने से पहले क्या उन्होंने आपको बताया था की वो क्यूँ कर रहे है ।क्या उन्होंने: हमेशा बताया था, कभी कभी बताया था, कभी नहीं बताया था? | Yes always told हाँ, हमेशा बताया था; Sometimes told कभी कभी बताया था;  No, never told नहीं, कभी नहीं बताया था; Don’t know नहीं पता;  No response/Refused to answer जवाब नहीं दिया/जवाब देने से मना किया | - Women struggled with the concept of having examinations and procedures explained to them before being conducted, we added an example but the construct itself is a challenge - Added “chek ap” [check up] alongside janch [check up] - Changed from “doctors and nurses” to “health workers” - Changed from tell/explain to tell - Changed from yes / no respose option to always / sometimes / never response option, with all three options read out |
| NA | NA | NA |  | Did any health worker tell you that you could say no to any of the examinations/checkups if you did not like them?  क्या स्वास्थकर्मियों में से किसीने आपको बताया था की आप कोई भी जांच/चेक के लिए मना कर सकती हैं यदि आपको वह जांच पसंद नहीं हो तो? | Yes हाँ; No नहीं; Don’t know पता नहीं;  No response/refused to answer जवाब नहीं दिया/जवाब देने मना किया | - New question added to attempt to measure consent |
| 9 | Did the doctors, nurses or other staff at the facility ask your consent before doing procedures and examinations on you?  क्या डाक्टर, नर्स या किसी अन्य स स्वास्थ्यकर्मी ने आपकी जांच कर ने से पहले आपकी सहमती ली थी? | Yes हाँ; No नहीं; Don’t know नहीं पता | 4 | Did the health workers ask/ take permission from you before doing vaginal examination or any other examination / checkup?  Did they: always ask, sometimes ask or never ask?  स्वास्थ्यकर्मी ने आपके योनि / बच्चा होने वाली जगह की जांच या आपकी कोई भी जांच करने से पहले क्या आपसे पूछा था या आपकी परमिशन ली थी? हमेशा पूछा था, कभी-कभी पूछा था, कभी नहीं पूछा थ? | Yes, always asked हाँ, हमेशा पूछा था; Sometimes asked कभी कभी पूछा था;  No, never asked नहीं, कभी नहीं पूछा था; Don’t know नहीं पता; No response/refused to answer उत्तर नहीं दिया/ देने से इंकार किया | - Women struggled with the concept of consent, we added an example but the construct itself is a challenge - Changed from “doctors, nurses, or other staff at the facility” to health workers - Provided two translations for “vaginal” [yoni and bacha hone waali jagah]and removed genital [guptang] because it was not well understood - Changed from “consent” [sahamathi] to “asked you” [aapse poocha tha] or “took your permission” [aapki parmishon li] |
| 10 | Please indicate if any of the following procedures were done without your permission (READ EACH OPTION AND CIRCLE RESPONSE)  कृपया बताएं क्या इनमें से कोई जांच बिना आपकी सहमती से हुई थी? [सारे विकल्प पढ़कर बताएं और लागू होने वाले विकल्पों को गोले में घेर दें] | No नहीं;  Yes, Tubal ligation हाँ, बंध्याकरण;  Yes, Abdominal palpation हाँ, पेट टटोलना;  Yes, genital examination हाँ, गुप्तांग की जांच;  Yes, Episiotomy [genital incision] हाँ, गुप्तांग में चीरा; Other (specify) अन्य (उल्लेख करें);  Don’t know नहीं पता | 5 | Can you tell me if any of these checks/procedures were done against your wish / will / without your permission? Was ___ done without your permission [READ EACH OPTION AND CIRCLE ALL APPLICABLE RESPONSES]  क्या आप हमें बतायेंगी कि इनमें से कोई भी जाँच/चेकअप आपकी इच्छा/मर्जी / परमिशन के बिना हुआ था?  क्या_______ आपकी इच्छा/मर्जी/ परमिशन के बिना हुआ था?  [सारे विकल्प पढ़कर बताएं और लागू होने वाले विकल्पों को गोले में घेर दें] | Abdominal check पेट की जाँच/ चेकअप; Vaginal examination योनि/बच्चा होने वाली जगह की जाँच;  Copper T कॉपर टी; Sterilization बंध्याकरण/ नसबंदी; Episiotomy [skip for c-section] योनि /बच्चा होने वाली जगह पे चीरा/ काटा गया था;  Other (specify) अन्य (उल्लेख करें);  Nothing was done without my permission कुछ भी मेरी इच्छा/ मर्जी से राज़ी से/ परमिशन के बिना नहीं हुआ; Don’t know नहीं पता;  No response/Refused to answer उत्तर नहीं दिया/ देने से इंकार किया | - Changed from “please tell” to “can you tell us” - Addedcheck up [chek ap] to check [janch] - Changed “consent” [sahamathi] to “wish” [ichchha] / “will” [marji]/ “permission” [parmishon] - Reordered response options from least to most invasive - Changed from “palpation” [tatolna] to “check” [janch / chek ap] - Added “copper T” because copper T insertion is sometimes done without women’s permission - Added a more common word for sterilization [“nasbandi”] to “sterilization” [banduakaran] - Changed episiotomy from “genital incision” [guptang mein chira] to “incision / cut in the vagina / baby place” [yoni / bachcha hone waali jagah pe chira / kata] - Noted in enumerator directions that more than one response is permitted |
| NA | NA | NA | 6 | At the time of delivery in the hospital who did the healthcare providers talk to about your care?  [READ ALL OPTIONS AND CIRCLE ALL THAT APPLY]  डिलीवरी के समय अस्पताल में स्वास्थ्य कर्मी ने आपकी देख भाल के बारे या आपकी जाँच / चेकअप के बारे में किस से बातचीत की? [सारे विकल्प पढ़कर बताएं और लागु होने वाले विकल्पों को गोले में घेर दें] | You आपसे; People in your family such as your husband or mother आपके परिवार वालों से जैसे आपके पति या माँ;  Other people you know like ASHA, friends आपके परिचय के कोई और लोग जैसे आशा, सहेली;  Someone you don't know, like other patients and strangers जिनको आप नहीं जानती जैसे दूसरे मरीज़ या अंजान व्यक्ति;  They didn’t speak to anyone किसी से बात नहीं की;  Don't know नहीं पता; No response / Refused to answer कोई जवाब नहीं | This new question that seeks to establish three things:   1. Whether the woman was spoken to about her care [a prerequisite to being involved in decisions, having procedures explained, giving consent, all of which are important RMC considerations] 2. Whether family members or other known people were spoken to but not the woman [because we found that in many cases providers only spoke to the husband or other companion] 3. Whether the woman’s care was discussed with strangers [a violation of confidentiality] |
| 11 | During your hospital stay for delivery, were you covered up with a cloth or blanket or screened with a curtain so that no outsider could see you?  प्रसव के लिए अस्पताल में रुकने के दौरान क्या आपको किसी कपड़े या कंबल से ढ़ककर रखा गया था या कसी प्रकार का पर्दा था ताकि कोई बाहरी व्यक्ति आपको देख ना सके? | Yes हाँ; No नहीं; Don’t know नहीं पता | 8 | At any point during your stay in hospital for delivery, were you left uncovered or in a room where others could see your body parts? Like, no curtain or screen was used to shield you, or doors and windows were left open so outsiders could see you.  डिलीवरी के लिए अस्पताल में रुकने के दौरान क्या आपको बिना ढ़के या फिर ऐसे कमरे में रखा गया जिससे आपके शरीर का कोई निजी भाग दूसरे लोग भी देख सकें? जैसे की ढंकने के लिए कोई पर्दा नहीं था, दरवाज़े और खिड़की खुली थी, जिससे बाहर का कोई व्यक्ति आपको देख सकता था. | Yes हाँ; No नहीं; Don’t know पतानहीं; No response / refused to answer जवाब नहीं दिया/ जवाब देने मना किया | - Changed directionality of the response from whether she was covered to whether she was ever left exposed - Changed “childbirth” [prasav] to “delivery [dilivri] - Changed from “outsider could see you” [baahari vyasthi dekh na sake] to “others could see private parts of your body” [aapake shareer ka koee nijee bhaag doosare log bhee dekh saken] - Added example |
| 12 | Were you allowed to have someone you wanted (outside of staff at the facility, such as family or friends) to stay with you during labor?  प्रसव के दौरान क्या आपको अनुमति थी कि स्वास्थकर्मी के अलावा आप अपने परिवार के किसी सदस्य या किसी जानने वाले को अपने साथ रख सकती है? | Yes हाँ; No नहीं; Don’t know नहींपता | 10 | Who was with you in the room when the baby was being born? [READ OUT OPTIONS AND CIRCLE ALL THAT APPLY]  जब बच्चा पैदा हो रहा था तब आपके साथ कमरे में कौन मौजूद था? [सारे विकल्प पढ़ कर बताएं और लागु होने वाले विकल्पों को गोले में घेर दें] | Mother मां; Mother-in-law सास; Sister/ sister-in-law बहन / भाभी; Husbandपति; Friend सहेली; ANM / nurse एएनएम / नर्स; ASHA आशा;  Doctor डॉक्टर; Traiditional birth attendant दाई;  Other (specify) अन्य (उल्लेख करें);  Alone अकेली थी;  Don’t know / not applicable नहीं पता/लागू नहीं; No response/Refused to answer उत्तर नहीं दिया/ देनेसे इंकार किया | - We re-wrote original questions #12, #13, #15, #16, #39 and #40 into new question #10 - New question #10 specifies who was in the room with them (because the original questions were unclear about whether to include companions who were around the hospital facility but not physically with the women) - New question #10 removes the concept of what is allowed/not allowed because respondents may not know and instead asks who was actually there - New question #10 removes father and father-in-law as potential companions because they are considered inapprorpaite resoponse options, and MAMTA because this health worker cadre does not exist in the area - In addition to enabling assessment of birth companionship, new question #10 also enables assessment of whether the woman was denied skilled professional health care by establishing if the doctor or nurse were in the room - New question #10 removes the distinction between labour and delivery and specifies who was with them when the baby was being born (delivery), since access to the delivery room is more likely to be restricted than access to the labour ward |
| 13 | Who was your companion during labor at the health facility? (READ OUT OPTIONS AND CIRCLE ALL THAT APPLY) प्रसव के दौरान आपके साथ स्वास्थ केंद्र पर कौन मौजूद था? (सारे विकल्प पढ़कर बताएं और लागु होने वाले विकल्पों को गोले में घेर दें।) | Mother/Father मां/बाप Mother-in-law सास  Father-in-law ससुर  Husband पति  Child बच्चे  Other relative अन्यरिश्तेदार  Friendसहेली  ANM/ ASHA/ MAMTA आशा/ एएनएम/ ममता  TBA/ Dai प्रशिक्षित दाई/दाई  Came alone अकेले आयी  Other (specify) अन्य (उल्लेख करें)  D on’t know नहीं पता 9898 | NA | NA | NA | - Removed, see new question #10 |
| 14 | During childbirth, were you allowed to get up, stand and walk?  क्या प्रसव के दौरान आपको उठने, खड़े होने या चलने की अनुमति थी? | Yes हाँ; No नहीं; Don’t know नहींपता; No response/Refusad to answer जवाब नहीं दिया/ जवाब देने से मना किया | 9 | During delivery, when you were feeling pain, were you allowed to get up and walk around?  डिलीवरी के दौरान जब आपको दर्द उठ रहे थे, तो क्या उस वक्त आपको उठने और अपने आस पास टहलने/ चलने फिरने की परमिशन/ अनुमति थी? | Yes हाँ; No नहीं; Don’t know पता नहीं; No response / refused to answer जवाब नहीं दिया/ जवाब देनेसे मना किया | - Changed “childbirth” [prasav] to “delivery [dilivri] - Added “when you were feeling pain” - Changed “get up, stand or walk” [uthane, khade hone ya chalane] to “get up and walk around” [uthane aur apane aasapaas tahalane / chalane phirane] because women found these synonyms clearer - Changed “permission” [parmishon] to “anumathi” [permission] |
| 15 | Were you allowed to have someone you wanted (outside of staff at the facility, such as family or friends) to stay with you during delivery?  प्रसव के दौरान क्या आपको अनुमति थी कि स्वास्थ कर्मी के अलावा आप अपने परिवार के किसी सदस्य या किसी जाननेवाले को अपने साथ रख सकती है? | Yes हाँ; No नहीं; Don’t know नहीं पता | NA | NA | NA | - Removed, see new question #10 |
| 16 | Who was your companion during delivery at the health facility? (READ OUT OPTIONS AND CIRCLE ALL THAT APPLY) प्रसव के दौरान आपके साथ स्वास्थ केंद्र पर कौन मौजूद था? (सारे विकल्प पढ़कर बताएं और लागु होने वाले विकल्पों को गोले में घेर दें।) | Mother/Father मां/बाप Mother-in-law सास  Father-in-law ससुर  Husband पति  Child बच्चे  Other relative अन्य रिश्तेदार  Friend सहेली  ANM/ ASHA/ MAMTA आशा/ एएनएम/ ममता  TBA/ Dai प्रशिक्षित दाई/ दाई  Came alone अकेले आयी  Other (specify) अन्य (उल्लेख करें)  Don’t know नहीं पता | NA | NA | NA | - Removed, see new question #10 |
| NA | NA | NA | 11 | When you were in the labour room, when the baby was about to come, what all positions did you take? [READ OUT OPTIONS AND CIRCLE ALL THAT APPLY]  जब आप डिलेवरी रुम में थी,जब बच्चा होने वाला था तो आप कौन-कौन से तरीके से लेटी या फिर खड़ी हुई थी? [सारे विकल्प पढ़कर बताएं और लागु होने वाले विकल्पों को गोले में घेर दें] | Lying on back पीठ के बल;  Standing खड़े; Squatting घुटने मोड़ के / उकडूँ बैठना / टॉलेट / लेटरीन करते समय जैसे बैठे है;  Other (specify) अन्य (उल्लेखकरें);  It was a caeserian section सिजेरियन सेक्शन (ऑपरेशन) हुआ था;  (skip next question)  (आगले प्रश्न पर जाए);  Don’t know /Do not remember नहीं पता/ याद नहीं; No response/Refused to answer उत्तर नहीं दिया/ देने से इंकार किया | - Because women could not understand the concept of other postioons for delivery beyond lying-on-back, we introduced this new question to expose them to the concept of multiple possible positions - This question then enabled us to ask whether or not they were free to chose the position they delivered in (which would almost always be on lying-on-back) - The place where babies are actually delivered is generally called the labour room not the delivery room, so we specified “in the labour room, when the baby was about to come” [laborroom mein thi, jab bacha hone waakaa tha] - We included multiple ways to say “squatting”: “squatting” [ukarhoo baitna]; “bent knee” [ghootane mord ke]; “toilet/latrine position” [tolet/letreen karthe samay jaise baithe hai] |
| 17 | Were you free to choose a position that was comfortable to you during your childbirth? [lit: Did your delivery happen in a position of your comfort or were you forbidden from being in the position of comfort? [क्या आप का प्रसव आप के अपने आराम के अनुसार की स्थिति में हुआ या अपने सहुलियत की स्थिति में रहने कि आपको मना ही थी? | Yes हाँ; No नहीं; Don’t know नहीं पता; No response/Refusal to answer जवाब नहीं दिया/ जवाब देने से मना किया | 12 | So you were ____ [REPEAT POSITION(S) MENTIONS IN PREVIOUS QUESTION]  Did you choose this/these position(s) or did they tell/make you take those position(s)?  तो आप ____ थी। [पिछले प्रश्न में चुने गए ऑप्शन को दोहराए] क्या ये आपने ख़ुद चुना था या किसिने आपको कहा या करवाया था? | I chose मैंनेचुना; I was made to take मुझसे करवाया; I chose and nurse also told मैंने चुना और नर्स ने भी बताया; Don’t know / not applicable नहीं पता/ लागू नही; No response/Refused to answer जवाब नहीं दिया/ जवाब देने मना किया | - Changed delivery [prasav] to “delivery” [dilivri] - Linked new #12 to to new #11 to try to increase respondent understanding of the concept of different positions - Changed from “forbidden to being in a position of comfort” [aapne sahooliyat ki sthithi mein rahne ki aapko manaahi thi] to “they made you do this” [kisee ke kahane par aisa kiya] - Changed response options |
| 19 | At any point during your stay for this delivery were you left unattended by health providers **when you needed care**?  प्रसव के दौरान क्या कभी ऐसा हुआ कि जब आप को **देखभाल की जरुरत थी** आपको स्वास्थकर्मीयों ने अकेला छोड़ दिया? | Yes हाँ; No नहीं; Don’t know नहींपता; No response/ Refused to answer जवाब नहीं दिया/ जवाब देने से मना किया | 13 | At any point during your stay for this delivery were you or your baby left unattended by health providers **when you needed care**?  इस डिलीवरी के लिए अस्पताल में ठहरने के दौरान क्या कभी ऐसा हुआ कि **देखभाल की जरुरत होने पर** भी आप को या आपके बच्चे को स्वास्थ्यकर्मियों ने अकेला छोड़ दिया हो? | Yes हाँ; No नहीं; Don’t know नहींपता; No response / Refused to answer जवाब नहीं दिया /जवाब देने से मना किया | - Changed delivery [prasav] to “delivery” [dilivri] - Added “or your baby” |
| 20 | When were you left unattended? (READ EACH OPTION ANDCIRCLE ALL THAT APPLY)  आपको कब अकेला छोड़ दिया गया? | While in labor प्रसव पीड़ा के दौरान;  While delivering प्रसव के दौरान;  While experiencing a complication परेशानी होने पर; After delivery प्रसव के बाद;  Baby after delivery प्रसव बाद बच्चे को; Other (Specify) अन्य (उल्लेख करें);  Don’t know नहीं पता | 14 | [IF YES TO#13] When were you or your baby left unattended? [READ EACH OPTION AND CIRCLE ALL THAT APPLY]  [यदि प्रश्न संख्या #13] का उत्तर हाँ हो: स्वास्थ्य कर्मियों ने कब आपको या आपके बच्चे को अकेला छोड़ दिया? [सारे विकल्प पढ़कर बताएं और लागु होने वाले विकल्पों को गोले में घेर दें।] | During delivery बच्चा होते समय; While experiencing a complication, difficulty or risk डिलीवरी में परेशानी या कठिनाई होते समय; You after delivery डिलीवरी के बाद आप को; Baby after delivery डिलीवरी के बाद बच्चे को; Other (Specify) अन्य (उल्ले खकरें);  Don’t know नहींपता | - Labour and delivery combined since the two concepts are not distinct - “While experiencing a complication” re-translated for clarity |
| 21 | Were you permitted to drink or eat while you were in labor?  क्या आप को प्रसव के दौरान कुछ खाने-पीने को दिया गया था या आपको कुछ खाने-पीने की अनुमति थी? | Yes हाँ; No नहीं; Don’t know नहीं पता; No response/ Refused to answer जवाब नहीं दिया/ जवाब देने से मना किया | 15 | At any point during your stay for this delivery were you prevented from eating or drinking when you were hungry/thirsty?  इस डिलीवरी के लिए अस्पताल में ठहरने के दौरान कभी भी जब आपको भूख या प्यास लगी थी तो क्या आपको खाने-पीने से रोका गया था? | Yes हाँ; No नहीं;  Don’t know नहीं पता; No response / Refused to answer जवाब नहीं दिया/ जवाब देने से मना किया | - Since it was hard for women to know what was permitted [anumati] or not, we changed it to asking if she was prevented/stopped [roka] from eating or drinking when hungry or thirsty |
| 22 | Were you or your family asked for a bribe or informal payment?  क्या आप से या आपके परिवार से किसी प्रकार की रिश्वत या पैसे की मांग हुई थी? | Yes हाँ; No नहीं; Don’t know नहीं पता | 16 | Were you or your family asked for a bribe or informal payment? Like, you were asked for money in the name of happiness.  क्या आप से या आप के परिवार से किसी प्रकार की रिश्वत या घूस या पैसे की मांग हुई थी? जैसे ख़ुशी के नाम पर आपसे पैसा माँगा गया हो। | Yes हाँ; No नहीं; Don’t know नहीं पता; No response / Refused to answer जवाब नहीं दिया/ जवाब देने से मना किया | - Added the local word for “bribe” [ghoos] - Added “Like you were asked for money in the name of happiness” [jaise khushee ke naam par aapase paisa maanga gaya ho] because many respondents are made to give money “in the name of happiness” and are not sure if they should report this as an informal payment |
| 23 | At any point during your stay for this delivery were you or your baby prevented from leaving this facility because you could not pay?  प्रसव के दौरान क्या आप को या आपके बच्चे को स्वास्थ केंद्र छोड़कर जाने से इसलिए रोका गया क्योंकि आप पैसे नहीं दे पा रही थी? | Yes हाँ; No नहीं; Don’t know नहीं पता | NA | NA | NA | - Removed because detainment at facilities was not a common form of disrespect and abuse in the region |
| 24 | How long after delivery did you or your baby have to stay because of your inability to pay?  प्रसव के बाद कितनी देर तक स्वास्थ केंद्र पर आप को या आपके बच्चे को पैसे देने की असमर्थता की वजह से रुकना पड़ा? | ____ Hours घंटे  ____ Days दिनों | NA | NA | NA | - Removed - Also we noted that women did not know why they stayed at facilities post-delovery for any set amount of time - Finally, enumerators found the response fields confusing – if seeking this data a clearer entry mechanism would be required (perhaps only days) |
| NA | NA | NA | 17 | Was there drinking water in the facility? Was drinking water always available, sometimes available or never available?  क्या अस्पताल में पीने के पानी की सुविधा थी? क्या पीने का पानी हर समय उपलब्ध था, कभी-कभी उपलब्ध था, कभी भी उपलब्ध नहीं था? | Always available हर समय उपलब्ध था; Sometimes available कभी-कभी उपलब्धथा; Never available कभी भी उपलब्ध नहीं था; Don’t know नहीं पता; No response/Refused to answer जवाब नहीं दिया/ जवाब देने मना से किया | - Added to separate drinking water (flitered water) from all other water - It is common for all homes, health facilities and businesses to have some drinking water but many do not have continuous available water for other purposes, such as cleaning - Included an always / sometimes / never response option to better capture cases where drinking water was sometimes unavailable but respondents were reluctant to say “no” when given a yes / no response option |
| 25 | Was there water in the facility?  क्या स्वास्थ्य केंद्र में पानी की सुविधा थी? | Yes हाँ; No नहीं; Don’t know नहीं पता | 18 | Apart from drinking water, was there water for cleaning in the hospital? Was water for cleaning always available, sometimes available or never available?  क्या अस्पताल में पीने के पानी के अलावा साफ़ सफ़ाई के लिए पानी की सुविधा थी? क्या साफ़ सफ़ाई के लिए पानी हर समय उपलब्ध था, कभी-कभी उपलब्ध था, कभी भी उपलब्ध नहीं था? | Always available हर समय उपलब्ध था; Sometimes available कभी-कभी उपलब्ध था; Never available कभी भी उपलब्ध नहीं था; Don’t know नहीं पता; No response / Refused to answer जवाब नहीं दिया/ जवाब देने से मना किया | - Specified that we are asking about non-drinking water, e.g. water for cleaning - Changed health centre [swasthya kendra] to hospital [aspitaal] - Changed to always / sometimes / never response option - All three response options to be read, to ensure that respondents are aware of the “sometimes” option |
| 26 | Was there electricity in the facility?  क्या स्वास्थ्य केंद्र में बिजली की सुविधा थी? | Yes हाँ; No नहीं; Don’t know नहीं पता | 19 | Was there electricity in the facility? Was electricity always available, sometimes available, never available?  क्या अस्पताल में बिजली की सुविधा थी? क्या बिजली हर समय उपलब्ध थी, कभी-कभी उपलब्ध थी, कभी भी उपलब्ध नहीं थी? | Always available हर समय उपलब्ध था; Sometimes available कभी-कभी उपलब्ध था; Never available कभी भी उपलब्ध नहीं था; Don’t know नहीं पता; No response / Refused to answer जवाब नहीं दिया/ जवाब देने से मना किया | - Changed to always / sometimes / never response option - All three response options to be read, to ensure that respondents are aware of the “sometimes” option |
| 27 | The doctors, nurses or other health care providers at the facility treated me with respect. By respect I mean being treated with the care and attention you deserve.  स्वास्थ्य केंद्र में डॉक्टर, नर्स और अन्य स्वास्थकर्मियों ने मेंरे साथ अच्छा व्यवहार किया। अच्छे व्यवहार से मेरा मतलब है कि मुझे जितना देखभाल और ध्यान की जरुरत थी वो मुझे मिला। | Strongly Agree पूरी तरह सहमत; Agree सहमत; Somewhat Agree थोड़ा सहमत; Somewhat disagree थोड़ा असहमत; Diagree असहमत; Strongly Disagree पूरी तरह असहमत | 22 | Did health care providers treat you with respect, meaning did they treat you with love and kindness? Did they treat you with respect always, sometimes, or never?  क्या स्वास्थ्यकर्मियों ने आपका मान-सम्मान किया/ आपको इज़्ज़त दिया? जैसेकी, आपकी प्यार के साथ देखभाल की। क्या उन्होंने हमेशा मान-सम्मान किया, कभी कभी मान-सम्मान किया, या कभी भी मान-सम्मान नहीं किया? | Always हर समय मान-सम्मान किया; Sometimes कभी कभी मान-सम्मान किया; Never कभी मान-सम्मान नहीं किया; Don’t know नहीं पता; No response/Refused to answer जवाब नहीं दिया/ जवाब देने से मना किया | - Changed “good behaviour” [achchhe vyavahaar] to “respect” [maan sammaan/izzat] - Removed “by respect I mean being treated with the care and attention you deserve” [achchhe vyavahaar se mera matalab hai ki mujhe jitana dekhabhaal aur dhyaan kee jarurat thee vo mujhe mila] because the literal translation, “by good behaviour I mean I got the care and attention I needed”, and the use of “good behaviour” led respondents to focus on whether they and their baby received the care needed for survival, not issues of respectful treatment - Added “Like, took care of you with love/kindness” [jaise ki, aapka pyaar ke saath dekhbhaal kiya] - Changed Likert response to always / sometimes / never, and all three options are read out |
| 28 | I felt that I could ask the doctors, nurses or other health care providers at the facility questions about my delivery. मुझे लगा कि मैं स्वास्थ्य केंद्र पर डॉक्टर, नर्स या किसी अन्य स्वास्थ कर्मीयों से अपने प्रसव के बारे में सवाल पूछ सकती थी। | Strongly Agree पूरी तरह सहमत; Agree सहमत; Somewhat Agree थोड़ा सहमत; Somewhat disagree थोड़ा असहमत; Diagree असहमत; Strongly Disagree पूरी तरह असहमत | 23 | Did you feel hesitation / fear / discomfort in asking the health care providers questions about your delivery? Did you always feel hesitation, sometimes feel hesitation, or never?  अपनी डिलीवरी के बारे में स्वास्थ्कर्मियों से सवाल पूछने में आपको कोई झिझक, हिचकिचाहट या परेशानी हुई? क्या आपको हर समय झिझक हुई, कभी कभी झिझक हुई, या फिर कभी भी झिझक नहीं हुई? | Always felt hesitation हर समय झिझक हुई; Sometimes felt hesitation कभी कभी झिझक हुई; Never felt hesitation कभी भी झिझक नहीं हुई; Don’t know नहीं पता; No response/Refused to answer जवाब नहीं दिया/ जवाब देने से मना किया | - Changed from “felt I could ask questions” to “felt hesitation / fear / discomfort in asking questions” to provide multiple example vocabulary words - This necessitated changing the directionality of the queston - Changed Likert response to always / sometimes / never, and all three options are read out |
| 29 | The doctors, nurses or other health care providers spoke to me in a language that I could understand. डॉक्टर, नर्स या अन्य स्वास्कर्मीयों ने मुझसे ऐसी भाषा में बात की जो मैं समझ सकती थी। | Strongly Agree पूरी तरह सहमत; Agree सहमत; Somewhat Agree थोड़ा सहमत; Somewhat disagree थोड़ा असहमत; Diagree असहमत; Strongly Disagree पूरी तरह असहमत | 24 | Did the healthcare providers speak to you in a language that you could understand? Did you always understand, sometimes understand or never understand?  क्या स्वास्थ्यकर्मियों ने आप से ऐसी भाषा या बोली में बात की जो आप समझ सकती थी ? क्या आप को उनकी भाषा/ बोली हर समय समझ आती थी? कभी कभी समझ आती थी? या कभी समझ नहीं आती थी? | Yes, always understoodहाँ, हर समय समझ आती थी; Sometimes understood कभी कभी समझ आती थी; No, never understood नहीं, कभी नहीं समझ आती थी; Don’t know नहीं पता; No response/Refused to answer जवाब नहीं दिया/ जवाब देने से मना किया | - Changed from “doctors, nurses or other healthcare providers” to “healthcare providers” - Changed from “language” [bhaasha] to “language or speech/dialect” [bhaasha ya bolee mein baat] - Changed Likert response to always / sometimes / never, and all three options are read out |
| 30 | The doctors, nurses or other health care providers at the facility involved me in decisions about my care. स्वास्थ्य केंद्र पर डॉक्टर, नर्स या अन्य स्वास्थ्यकर्मियों ने मेरे स्वास्थ से संबंधित बातों के लिए मुझसे भी राय ली। | Strongly Agree पूरी तरह सहमत; Agree सहमत; Somewhat Agree थोड़ा सहमत; Somewhat disagree थोड़ा असहमत; Diagree असहमत; Strongly Disagree पूरी तरह असहमत | NA | NA | NA | - We were unable to develop a question that worked for this population - New question #6 identifies who the doctors spoke to, but is not truly able to measure the construct of being involved in decision-making |
| 31 | The doctors, nurses or other health care providers at the facility asked my permission or consent before undertaking clinical procedures.  स्वास्थ केंद्र पर डॉक्टर, नर्स या अन्यस्वास्थ्य कर्मियों ने मेरी किसी भी तरह की जांच प्रक्रिया से पहले मुझसे सहमति ली। | Strongly Agree पूरी तरह सहमत; Agree सहमत; Somewhat Agree थोड़ा सहमत; Somewhat disagree थोड़ा असहमत; Diagree असहमत; Strongly Disagree पूरी तरह असहमत | NA | NA | NA | - Removed, see revised questions#3 and #4 |
| 32 | The doctors, nurses or other health care providers at the facility explained exams and procedures to me before performing them.  स्वास्थ केंद्र पर डॉक्टर, नर्स या अन्य स्वास्थ्कर्मियों ने मेरी किसी भी जांच प्रक्रिया को करने से पहले मुझे उसके बारे में पूरी तरह बताया/ समझाया। | Strongly Agree पूरी तरह सहमत; Agree सहमत; Somewhat Agree थोड़ा सहमत; Somewhat disagree थोड़ा असहमत; Diagree असहमत; Strongly Disagree पूरी तरह असहमत | NA | NA | NA | - Removed, see revised #2 and #6 |
| 33 | The doctors, nurses or other health care providers explained the results to me from exams and clinical procedures undertaken.  डॉक्टर, नर्स या अन्य स्वास्थ्यकर्मियों ने म मुझ पर की गई हर जांच प्रक्रिया के परिणामों के बारे में मुझे बताया। | Strongly Agree पूरी तरह सहमत; Agree सहमत; Somewhat Agree थोड़ा सहमत; Somewhat disagree थोड़ा असहमत; Diagree असहमत; Strongly Disagree पूरी तरह असहमत | NA | NA | NA | - Removed, strong overlap with #2 and #6 |
| 34 | My personal health information will be kept confidential at this health facility. इस स्वास्थ केंद्र पर मेरी निजी स्वास्थ्य संबंधी जानकारी को गोपनीय रखा जाएगा। | Strongly Agree पूरी तरह सहमत; Agree सहमत; Somewhat Agree थोड़ा सहमत; Somewhat disagree थोड़ा असहमत; Diagree असहमत; Strongly Disagree पूरी तरह असहमत | NA | NA | NA | - Removed, see revised question #6 and #7 |
| 35 | I was left alone in the facility when I needed assistance from the doctors, nurses or other staff. स्वास्थ केंद्र पर जब मुझे डॉक्टर, नर्स या किसी अन्य स्वास्थ्कर्मियों के मदद की जरुरत थी तब उन्होनें मुझे अकेला छोड़ दिया। | Strongly Agree पूरी तरह सहमत; Agree सहमत; Somewhat Agree थोड़ा सहमत; Somewhat disagree थोड़ा असहमत; Diagree असहमत; Strongly Disagree पूरी तरह असहमत | NA | NA | NA | - Removed, see revised question #13 |
| 36 | The health care provider helped me to try different delivery positions. स्वास्थ्यकर्मियों ने प्रसव के दौरान मुझे अपने अनुकुल स्थितीयों को अपनाने में मेरी मदद की। | Strongly Agree पूरी तरह सहमत; Agree सहमत; Somewhat Agree थोड़ा सहमत; Somewhat disagree थोड़ा असहमत; Diagree असहमत; Strongly Disagree पूरी तरह असहमत | NA | NA | NA | - Removed, see revised questions #9, #11 and #12 |
| 37 | The doctors, nurses or other health care providers at the facility did everything they could to help control my pain. स्वास्थ्य केंद्र पर डॉक्टर, नर्स या अन्य स्वास्थ्यकर्मियों ने मेरे दर्द को रोकने का हर संभव प्रयास किया। | Strongly Agree पूरी तरह सहमत; Agree सहमत; Somewhat Agree थोड़ा सहमत; Somewhat disagree थोड़ा असहमत; Diagree असहमत; Strongly Disagree पूरी तरह असहमत | 25 | Did the health workers do everything they could to help reduce your discomfort and pain?  क्या आपकी तकलीफ़ और दर्द को कम करने के लिए स्वास्थकर्मियों ने वह सब किया जो वो कर सकते थे? | Yes हाँ; No नहीं; Don’t know नहीं पता;  No response / Refused to answer जवाब नहीं दिया/ जवाब देने से मना किया | - Changed from Likert to yes / no response - Translation improved |
| 38 | The doctors, nurses, or health care providers explained to me why they were giving me medicine. डॉक्टर, नर्स या अन्य स्वास्थ्यकर्मियों ने मुझे समझाया कि वो मुझे दवाईयां क्यों दे रहे थे। | Strongly Agree पूरी तरह सहमत; Agree सहमत; Somewhat Agree थोड़ा सहमत; Somewhat disagree थोड़ा असहमत; Diagree असहमत; Strongly Disagree पूरी तरह असहमत | NA | NA | NA | - Removed, see revised question #2 and #6 |
| 39 | When I was speaking to doctors, nurses or other health care providers at the facility, other people not involved in my care could hear what we were discussing. जब मैं स्वास्थ्य केंद्र पर डॉक्टर, नर्स या किसी अन्य स्वास्थ्यकर्मियों से बात कर रही थी तब वहां मौजूद दूसरे अन्य व्यक्ति भी हमारी बातें सुन सकते थे। | Strongly Agree पूरी तरह सहमत; Agree सहमत; Somewhat Agree थोड़ा सहमत; Somewhat disagree थोड़ा असहमत; Diagree असहमत; Strongly Disagree पूरी तरह असहमत | NA | NA | NA | - Removed, see revised question #6 &#7 |
| 40 | I was allowed to have the birth companion of my choice present during labor. प्रसव पीड़ा के दौरान मैं अपने किसी नजदीकी व्यक्ति को अपने साथ रख सकती थी। | Strongly Agree पूरी तरह सहमत; Agree सहमत; Somewhat Agree थोड़ा सहमत; Somewhat disagree थोड़ा असहमत; Diagree असहमत; Strongly Disagree पूरी तरह असहमत | NA | NA | NA | - Removed, see revised question #10 |
| 41 | I was allowed to have the birth companion of my choice present during delivery. `प्रसव के दौरान मैं अपने किसी नजदीकी व्यक्ति को अपने साथ रख सकती थी। | Strongly Agree पूरी तरह सहमत; Agree सहमत; Somewhat Agree थोड़ा सहमत; Somewhat disagree थोड़ा असहमत; Diagree असहमत; Strongly Disagree पूरी तरह असहमत | NA | NA | NA | - Removed, see revised question #10 |
| 42 | The health facility washrooms were clean.  स्वास्थ्य केंद्र के शौचालय/ स्नानागार में सफाई थी। | Strongly Agree पूरी तरह सहमत; Agree सहमत; Somewhat Agree थोड़ा सहमत; Somewhat disagree थोड़ा असहमत; Diagree असहमत; Strongly Disagree पूरी तरह असहमत | 20 | Would you say the health facility washrooms were very clean, somewhat clean and somewhat dirty or very dirty?  आप के हिसाब से अस्पताल के शौचालय की सफाई कैसी थी? क्या वो बहुत साफ-सुथरा था, थोड़ा साफ या थोड़े गंदा या फिर बहुत ही ज्यादा गंदा था? | Very clean बहुत साफ़;  Somewhat clean / somewhat dirty थोड़ा साफ़/ थोड़ा गंदा;  Very dirty बहुत गंदा; There were no washrooms in the facility शौचालय नहीं थे;  I did not have a need to use the washroom मैंने शौचालय इस्तेमाल नहीं किया;  Don’t know नहीं पता; No response/Refused to answer जवाब नहीं दिया/ जवाब देने से मना किया | - Removed Likert response - Provided three response options, which are all read out |
| 43 | The general health facility environment was clean. स्वास्थ्य केंद्र का वातावरण/ माहौल स्वच्छ था। | Strongly Agree पूरी तरह सहमत; Agree सहमत; Somewhat Agree थोड़ा सहमत; Somewhat disagree थोड़ा असहमत; Diagree असहमत; Strongly Disagree पूरी तरह असहमत | 21 | Overall, would you say the health facility was very clean, somewhat clean and somewhat dirty or very dirty?  आपके हिसाब से कुलमिलाकर अस्पताल में साफ-सफाई कैसी थी? क्या अस्पताल बहुत साफ-सुथरा था, थोड़ा साफ या थोड़े गंदा या फिर बहुत ही ज्यादा गंदा था? | Very clean बहुत साफ़; Somewhat clean / somewhat dirty थोड़ा साफ़/ थोड़ा गंदा;  Very dirty बहुत गंदा; Don’t know नहीं पता; No response/Refused to answer जवाब नहीं दिया/ जवाब देने मना से किया | - Removed Likert response - Provided three response options, which are all read out |
| 44 | There were enough staff in the health facility to care for me.  स्वास्थ्य केंद्र पर मेरी देखभाल के लिए काफी स्वास्थ्यकर्मी मौजूद थे। | Strongly Agree पूरी तरह सहमत; Agree सहमत; Somewhat Agree थोड़ा सहमत; Somewhat disagree थोड़ा असहमत; Diagree असहमत; Strongly Disagree पूरी तरह असहमत | NA | NA | NA | - Removed because women found it difficult to assess the idea of “enough” staff. They felt that if there was anyone there it was sufficient. - In addition, “care” [dekhbhal] is provided by family members; injections and other medical procedures are done by staff |
| 45 | I am satisfied with my overall experience during my most recent delivery visit.  कुलमिलाकर अपने हाल ही के प्रसव के दौरान स्वास्थ्य केंद्र की सुविधाओं और अपने अनुभवों से मैं संतुष्ट हूँ. | Strongly Agree पूरी तरह सहमत; Agree सहमत; Somewhat Agree थोड़ा सहमत; Somewhat disagree थोड़ा असहमत; Diagree असहमत; Strongly Disagree पूरी तरह असहमत | 31 | How good was your overall experience during your delivery? Very good, somewhat good / somewhat bad, very bad?  कुलमिलाकर अस्पताल में डिलीवरी का अनुभव आपका कैसा रहा? बहुत ही अच्छा, थोड़ा अच्छा या थोड़ा खराब या फिर बहुत ही खराब? | Very good बहुत अच्छा; Somewhat good/somewhat bad थोड़ा अच्छा/ थोड़ा ख़राब;  Very bad बहुत ख़राब; Don’t know नहीं पता; No response/ Refused to answer जवाब नहीं दिया/ जवाब देने से मना किया | - Removed Likert response - Provided three response options, which are all read out |
| 46 | If I had more children in the future, I would delivery them at the same health facility.  अगर भविष्य में मुझे और बच्चे हुए तो मैं अपने प्रसव के लिए उसी स्वास्थ्य केंद्र पर जाउंगी। | Strongly Agree पूरी तरह सहमत; Agree सहमत; Somewhat Agree थोड़ा सहमत; Somewhat disagree थोड़ा असहमत; Diagree असहमत; Strongly Disagree पूरी तरह असहमत | NA | NA | NA | - Removed because respondents focused on their capacity to afford or access other options in the future or because respondents focused on their likelihood to have another child, rather than on issues related to quality of care |
| 47 | I would recommend the place of my most recent delivery visit to other women.  अभी हालही में जिस स्वास्थ्य केंद्र पर मेरा प्रसव हुआ मैं वहां दूसरी औरतों को भी जाने की राय दूँगी. | Strongly Agree पूरी तरह सहमत; Agree सहमत; Somewhat Agree थोड़ा सहमत; Somewhat disagree थोड़ा असहमत; Diagree असहमत; Strongly Disagree पूरी तरह असहमत | NA | NA | NA | - Removed because respondents focused on their capacityto make recommendations, speak to or influence other women, rather than on issues related to quality of care |
| 48 | All health workers treat patients equally  सभी स्वास्थ्यकर्मी सारे मरीजों या रोगीयों से एकसमान / एक जैसा बर्ताव करते हैं | Strongly Agree पूरी तरह सहमत; Agree सहमत; Somewhat Agree थोड़ा सहमत; Somewhat disagree थोड़ा असहमत; Diagree असहमत; Strongly Disagree पूरी तरह असहमत | 26 | Do you think all healthcare providers treat patients equally?  क्या आपको लगता है कि सभी स्वास्थ्यकर्मी सारे मरीजों या रोगीयों से एकसमान / एक जैसा बर्ताव करते हैं? | Yes हाँ; No नहीं;  Don’t know पता नहीं;  No response / refused to answer जवाब नहीं दिया/ जवाब देने मना से किया | - Removed Likert response - Provided three response options, which are all read out |
| 49 | When you needed help, did you feel the doctors, nurses or other staff at the facility paid attention?  जब आपकी मदद जी ज़रूरत थी, तब आपको क्या ये महसूस हुआ था के अस्पताल में मौजूद डॉक्टर, नर्स या अन्य स्वस्थ्यकर्मियों ने आप पर ध्यान दिया? | Strongly Agree पूरी तरह सहमत; Agree सहमत; Somewhat Agree थोड़ा सहमत; Somewhat disagree थोड़ा असहमत; Diagree असहमत; Strongly Disagree पूरी तरह असहमत | NA | NA | NA | - Removed. - Revised question #13 assesses the same construct of being left unattended when needing care |
| 51 | During your hospital stay, did health providers ever discuss your personal private health information in a way that others could hear?  अस्पताल में रुकने के दौरान क्या किसी भी स्वास्थकर्मी ने आपके स्वास्थ की निजी और गोपनीय जानकारी इसतरहसे बताई कि वहां मौजूद अन्य लोगों को भी मालूम हो जाए? | Yes हाँ; No नहीं; Don’t know नहीं पता | 7 | During your hospital stay for delivery, did health providers ever discuss your results in front of unknown people? Like, reading your report loudly, so that others might hear it.  जब आपको डिलीवरी के लिए अस्पताल में रखा गया था तो क्या स्वास्थ्यकर्मियों ने कभी भी आपकी जांच या रिपोर्ट के नतीजों को अंजान लोगों के सामने बताया? जैसे कि आपकी रिपोर्ट को इतनी जोर-जोर से पढi (ऊँची आवाज में पढना) जिससे वहां मौजूद दूसरे लोग सुन सकें? | Yes हाँ; No नहीं;  Don’t know पता नहीं; No response / refused to answer जवाब नहीं दिया/ जवाब देने से मना किया | - Changed “private and confidential information about your health” [swaasth kee nijee aur gopaneey jaanakaaree] to “results from reports” [janch ya riport nateeji] - Changed “other people present” [mozood anya logon] to “unknown people” [anzaan logoon] - Added example |
